# An alternative technique for organelle genome recovery in diatoms using culture-independent, minimal-cell whole genome amplification

**DOI:** 10.7717/peerj.20767

**Published:** 2026-02-25

**Authors:** Aimee Caye G. Chang, Mailor W.W. Amaral, Kyle Keepers, Catherine Ikudaisi, Megan Greenwood, Jingchun Li, Sarah E. Hamsher, Scott R. Miller, John Patrick Kociolek

**Affiliations:** 1 Museum of Natural History and Department of Ecology and Evolutionary Biology, University of Colorado at Boulder, Boulder, CO, United States of America; 2 Department of Biological Sciences, College of Science, University of Santo Tomas, Manila, National Capital Region, Philippines; 3 Department of Biology and Annis Water Resources Institute, Grand Valley State University, Allendale and Muskegon, MI, United States of America; 4 Division of Biological Sciences, University of Montana, Missoula, MT, United States of America

**Keywords:** Algae, Plastomes, Mitogenomes, *Campylodiscus*, Plagiotropis, Organelles, mcWGA

## Abstract

**Background:**

This study presents an alternative method in diatom genomics using two raphid diatoms—*Campylodiscus clypeus* and *Plagiotropis lepidoptera*—whose organellar genome characteristics have remained unexplored due to cultivation constraints. Only a small fraction of the estimated 200,000 diatom species has been cultured in the laboratory. This research showcases the use of minimal-cell genomics as a viable alternative for studying diatoms and other eukaryotic microorganisms that do not respond well to traditional laboratory culture methods.

**Methods:**

Initial attempts to culture *C. clypeus* and *P. lepidoptera* were unsuccessful, hindering the acquisition of genomic data. To overcome these challenges, we employed minimal-cell whole genome amplification (mcWGA) techniques for two uncultured species, followed by metagenomic sequencing and assembly. This enabled direct genomic recovery from minimally isolated and pooled cells, eliminating the need for cultivation.

**Results:**

Using mcWGA approach, we successfully obtained the complete chloroplasts and mitochondrial genomes of *C. clypeus* and *P. lepidoptera* using only 8–12 viable cells isolated from fresh environmental samples. The plastome size of *C. clypeus* was 143,367 bp and mitogenome size was 46,274 bp, while *P. lepidoptera* has plastome and mitogenome sizes of 116,161 bp and 49,356 bp, respectively. The data generated provides a valuable resource for further research, highlighting the importance of culture-independent techniques in microbial genomics.

## Introduction

Diatoms are microalgae that play a key role in aquatic ecosystems, contributing to global carbon cycling and serving as indicators of environmental changes such as water quality and climate shifts (Benoiston et al., 2017; B-Béres et al., 2023). Organelle genome studies of diatoms provide valuable insights into their biology and taxonomy, including their ability to perform photosynthesis and adapt to varying environmental conditions (Mock & Medlin, 2012). However, obtaining complete diatom genomes is often challenging due to their complex genome structures and the difficulties associated with cultivating them in a laboratory. Consequently, researchers are exploring alternative approaches, such as metagenomics, single-cell sequencing, and transcriptomics, to study diatom genomes (Hamilton, Lefebvre & Bull, 2015; Knjaz et al., 2024; Sieracki et al., 2019). These methods allow scientists to analyze diatom genomes in mixed communities or from single cells, overcoming limitations posed by traditional culturing techniques. Such advancements enhance our ability to understand diatom functions and their potential applications in areas like biotechnology and environmental conservation.

Culturing diatoms presents numerous challenges due to their specific and often demanding environmental requirements in vitro. Many diatoms require precise conditions for light, temperature, pH, salinity, and nutrient availability, which can be difficult to replicate and maintain in laboratory settings (Iwasaki et al., 2021; Tavčar Verdev & Dolinar, 2025). Some diatoms have slow growth rates, others can be small and hard to isolate, other algal taxa, especially cyanobacteria, can contaminate cultures, and the reliance on specialized media and techniques for isolating cells further complicate the cultivation process to achieve unialgal cultures (Tavčar Verdev & Dolinar, 2025). These factors contribute to the limited availability of genomic data for many diatom species, as successful culturing is a crucial step for obtaining high-quality and quantity DNA for sequencing and other molecular studies (Kollár, Kopalová & Kohler, 2025; Nenasheva et al., 2025). Of the approximately 75,000 diatom taxa described (Kociolek et al., 2025), only around 6,400 diatom strains are currently maintained in culture (Vaulot et al., 2024). However, the actual number of unique taxa may be lower due to potential redundancies in strain names. But regardless, this represents a small fraction of the estimated 100,000-200,000 diatom species (Mann & Droop, 1996; Mann & Vanormelingen, 2013; Wang et al., 2022), highlighting the difficulty of culturing a significant portion of diatom biodiversity (Kollár, Kopalová & Kohler, 2025). Given the increasing prevalence and importance of using multiple genetic markers for resolving diatom taxonomy to the species level (Hamsher et al., 2019), a solution to the widespread difficulty of culturing diatoms for sequencing is desperately needed.

While advancements in sequencing technologies have facilitated genomic studies for many different types of algae (National Center for Biotechnology Information (NCBI), 2025c), comprehensive DNA sequence data for diatoms are limited compared to other photosynthetic organisms. The paucity of genomic data in diatoms applies to our focal taxa, *C. clypeus* and *P. lepidoptera*. Existing studies often focus on morphological and ecological aspects (Kamberović et al., 2024; Wang et al., 2024), leaving genetic insights underexplored. For the genus *Campylodiscus*, 153 gene sequences are available in NCBI (including *18S* rRNA, *28S* rRNA, *rbcL*, *psbC*, and *cob* genes), representing approximately 17 taxa. In contrast, data for the genus *Plagiotropis* are more limited, with only nine sequences deposited to date, covering just two taxa and comprising *18S* rRNA, *28S* rRNA, *rbcL*, and *psbC* genes. As a result, there is a pressing need for more genomic research to better understand these taxa as neither of these species has documented complete organellar genomes. *C. clypeus* is the generitype of the genus (Poulícbreve; ková & Jahn, 2007) while there are differing interpretations of the phylogenetic position of the genus *Plagiotropis* (Nézan et al., 2018).

In this paper, we report the successful use of culture-independent, mcWGA approach to assemble the whole chloroplast genome, as well as one complete mitochondrial genome and one partial mitogenome, of two raphid diatoms, *C. clypeus* and *P. lepidoptera*, which represent the first documented whole genomes from their respective genera. We were also able to recover the nuclear ribosomal *18S* and *28S* RNA sequences from our assemblies, demonstrating that single-cell WGA can recover all traditional taxonomic barcoding sequences, in addition to the entire organellar genomes and portions of the nuclear genome as well. We selected two raphid diatom genera, *Campylodiscus* and *Plagiotropis*, as study systems. While raphid diatoms in general are comparatively well sampled at the organellar genome level, complete chloroplast and mitochondrial genomes are still lacking for these genera. Expanding representation to include *Campylodiscus* and *Plagiotropis* therefore provides valuable new resources for genus-level comparative genomics and evolutionary studies. Both taxa were also abundant and morphologically distinctive in our samples, facilitating confident identification and pooling of multiple cells for sequencing. These features made them well suited to demonstrate the use of our minimal-cell approach. Our findings show that the mcWGA technique described in this paper is a feasible and effective alternative method for genomic studies of diatoms that are challenging to culture.

## Materials & Methods

### Sample collection and strain identification

Samples used in this study were collected from two locations in California. *C. clypeus* was obtained from an oasis in Joshua Tree National Park (coordinates: 33.881866°N, −115.900650W) on April 7th, 2024, around 10:00 AM, under collection permit #JOTR-2023-SCI-0012 issued by the National Park Service. *P. lepidoptera* was collected from a coastal lagoon on Vance Avenue, Humboldt County, California (coordinates: 40.828588°N, −124.171999°W) on August 31st, 2024, around 4:00 PM, which did not require a collection permit. Samples were transported to the laboratory within 72 h of collection for immediate processing.

Initial strain identification using a light microscope was performed to confirm the presence of *C. clypeus* and *P. lepidoptera* in the environmental samples. During this process, a subset of samples was transferred to separate tubes for final strain identification.

For taxon identification, samples were cleaned with nitric acid, rinsed, settled for 24 h and then rinsed again five times. The cleaned material was airdried onto cover slides. For light microscopy, the dried material was dried onto coverslips and mounted onto glass slides with Hyrax. Light microscopes observations were made on an Olympus BX-51 light microscope outfitted with differential interference contrast (DIC). Observations were made with 60X and 100X oil-immersion objectives. Digital images were captured with a DP-71 digital camera. Permanent glass microscope slides are deposited as vouchers 653099 *(Campylodiscus clypeus*) and 653100 *(Plagiotropis lepidoptera*) in the Kociolek Diatom Collection at the University of Colorado, Boulder (COLO).

For scanning electron microscopy (SEM), coverslips containing the dried material were attached to aluminum stubs with double-sided carbon tape. The stubs were then sputter-coated with 4 nm of Pt and viewed on a Hitachi SU−3500 VP SEM at an accelerating voltage of 15 kV and at a working distance of 5.5 mm at the Colorado Shared Instrumentation in Nanofabrication and Characterization (COSINC) facility at The University of Colorado, Boulder.

### Unsuccessful culturing of *Campylodiscus clypeus* and *Plagiotropis lepidoptera*

Attempts were made to cultivate *C. clypeus* and *P. lepidoptera* using both f/2 (Guillard, 1975) and WC medium (Guillard & Ryther, 1962) supplemented with nitrogen (WC +N), following standard diatom culturing protocols including serial dilutions and single cell isolations described by Vaulot et al. (2024). Cells were then incubated at ambient room temperature (∼24 °C) without adjustement to the water temperature, under a natural light-dark cycle. However, despite repeated efforts, neither taxon showed sustained growth or division in culture. *P. lepidoptera* showed initial signs of growth but eventually died in culture. Although *C. clypeus* and *P. lepidoptera* were grown in f/2 medium and WC medium supplemented with nitrogen, trace metals, and vitamins, long-term cultivation of these species was challenging probably due to potential species-specific nutritional requirements, such as obligatory symbioses. Other studies (Ruck et al., 2016; Sabir et al., 2018) successfully cultured some strains of *Plagiotropis*, but our attempts to culture it were unsuccessful, likely due to the lack of specialized growth chambers and differences in the composition of the culture media. The failure to establish cultures of these taxa using conventional methods prompted us to develop an alternative strategy, enabling us to recover genomic data directly from individual or minimally pooled cells without the need for prior cultivation.

### Isolation of viable diatom cells

Healthy and intact diatom cells were isolated from freshly collected environmental samples to ensure viability for successful genome amplification and recovery. Ten cells were isolated for *C. clypeus* and 12 cells for *P. lepidoptera*. To address challenges encountered during the mitogenome assemblies, an additional round of amplification was carried out for *P. lepidoptera* to get long-read sequencing data. For this, eight cells were isolated from the same sample bags used in the previous extraction. Unfortunately for *C. clypeus*, no more viable cells were recovered for further extraction and sequencing, and thus no long-read sequence was produced for *C. clypeus*.

**Figure 1:**
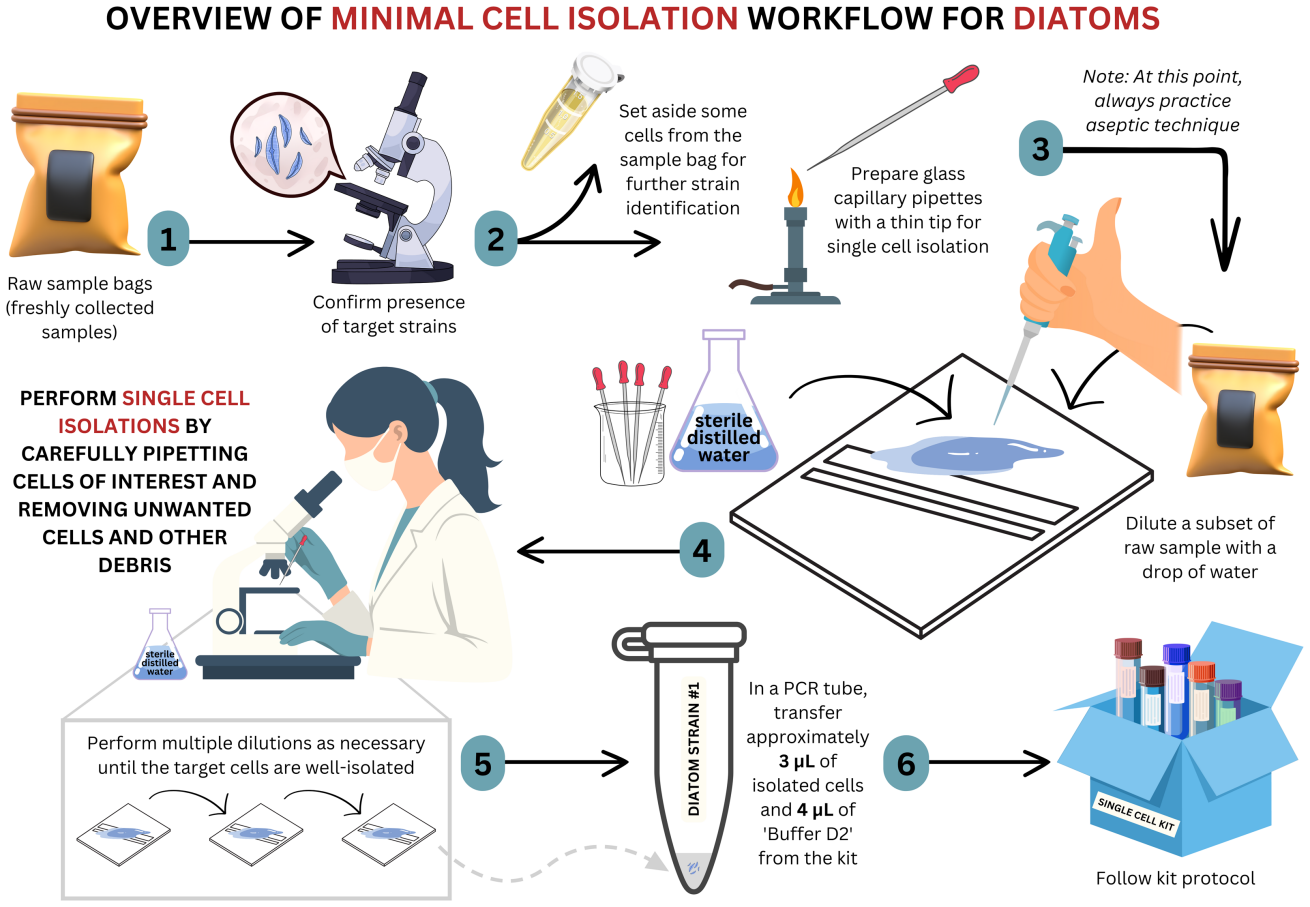
Workflow for mcWGA technique. An overview of the cell isolations and minimal-cell whole genome amplification (mcWGA) workflow developed in this study facilitating diatom isolation without the need for culturing.

To minimize potential contamination and ensure cell purity, the samples were subjected to a series of dilution steps (Fig. 1). Cells were diluted multiple times in sterile, filtered distilled water to isolate single diatom cells as effectively as possible. This was performed until no unwanted cells and debris were seen under the microscope. This isolation process ensured that the cells remained viable and reduced the presence of potential contaminants, facilitating reliable downstream genomic analysis. A step-by-step video guide of the cell isolations and mcWGA method for diatoms is provided as a Supplementary File (https://doi.org/10.6084/m9.figshare.29473958).

### Whole genome amplification

Whole genome amplification (WGA) was performed using the Repli-G Single Cell Kit (Qiagen, Hilden, Germany) according to the manufacturer’s instructions. This kit employs multiple displacement amplification (MDA) technology with Phi29 DNA polymerase and random hexamer primers to amplify all genomic DNA present in the sample. Additional details on the kit technology and primer system are provided in Meier et al. (2014). Freshly collected viable cells were isolated into 0.2-ml PCR tubes, lysed with the denaturation buffer provided in the kit, and incubated at 65 °C for 10 mins. The amplification master mix was then prepared and added to the samples, followed by incubation at 30 °C for 8 h to facilitate isothermal amplification. The reaction was terminated by heating to 65 °C for 3 mins, and the amplified DNA was stored at −20 °C until further analysis. This amplification targeted the entire genomic DNA of the cells, including nuclear and organellar genomes. Organellar sequences were identified and filtered during downstream bioinformatic analyses. The concentration of the amplified DNA was measured using a Qubit fluorometer (Thermo Fisher Scientific) following the manufacturer’s protocol to ensure accurate quantification.

### Sequencing, assembly and annotation

Amplified DNA samples were sent to SeqCoast Genomics (Portsmouth, NH, USA) for short-read sequencing on the Illumina NextSeq2000 platform. The company prepared the samples for whole genome sequencing using the Illumina DNA Prep tagmentation kit (#20060059) with Illumina Unique Dual Indexes and then performed a clean and concentrate step to remove residual PCR components before library preparation and sequencing, following their standard protocols.

Additional Nanopore long-read sequencing data were incorporated into the assembly to resolve the highly repetitive region of the *P. lepidoptera* mitogenome. DNA samples were prepared for whole-genome sequencing using the Oxford Nanopore Technologies Native Barcoding Kit (SQK-NBD114), with Long Fragment Buffer to enhance read lengths. Sequencing was conducted on the PromethION 2 Solo platform using an R10 version FLO-PRO114M Flow Cell at a translocation speed of 400 bps. Base calling was performed on the GridION using the super-accurate basecalling model with barcode trimming enabled. Data were returned as FASTQ files for subsequent analysis.

The raw reads were trimmed and filtered using Trimmomatic v0.39 (Bolger, Lohse & Usadel, 2014) to ensure high-quality input data prior to assembly. Genome assembly for the short-read libraries of *C. clypeus* was performed using metaSPAdes v3.11.1 (Nurk et al., 2017) optimized for de novo assembly of sequencing data generated from the Illumina platform. In the case of *P. lepidoptera*, a hybrid assembly was generated in metaSPAdes using the Illumina short-reads combined with the Nanopore long reads. Genome assembly statistics on all assemblies were generated using QUAST (Gurevich et al., 2013), which provided detailed metrics such as the number of contigs, N50 value, and total assembly size to evaluate the quality of the assembled genome. Long-read data quality was assessed using Nanoplot (De Coster et al., 2018). Organellar scaffolds of interest were identified using a BlastN search of the assembled contigs against a relevant database of proteins that should be contained within the respective organellar genomes. For the *C. clypeus* plastome, we used the reference sequence <*Epithemia pelagica*; accession OX459761.1 >. For *C. clypeus* mitogenome, we used <*Epithemia pelagica*; accession OX337243.1 >. For *P. lepidoptera* plastome, <*Pleurosigma intermedium*; accession OL415008.1 >was used. For its mitogenome, we used <*Pleurosigma* sp.; accession MW861541.1 >. The BlastN searches facilitated the filtering of the assembled sequences against known reference for diatom plastomes and mitogenomes.

Putative contigs were first identified and connected through a tiling process, in which reads were iteratively extended until overlapping regions allowed contig joining. Circularization was achieved by extending sequence ends until the genomes formed a continuous loop. To validate assembly quality, reads were aligned to the assembled genomes using *bwa mem* (Li & Durbin, 2009). The resulting alignments were sorted and indexed using SAMtools *tview* (Li et al., 2009). Variant calling was performed with *bcftools mpileup* and *bcftools call* (Narasimhan et al., 2016) to identify single nucleotide polymorphisms (SNPs) and assess consensus accuracy. Variants were filtered to retain high-quality calls (minimum quality score > 100), providing confidence in the assembly sequence. Post-assembly error correction was performed by updating the consensus sequence based on the filtered variant calls, ensuring high accuracy of the final genome assemblies.

Organellar genome annotation was performed using GeSeq (Tillich et al., 2017), validated on Sequin v10.3 (NCBI) and visualized using OGDRAW (Greiner, Lehwark & Bock, 2019) in Chlorobox (https://chlorobox.mpimp-golm.mpg.de), a specialized web tool for initiating an annotation of chloroplast and mitochondrial genomes. The boundaries of protein-coding sequences, tRNAs, and rRNAs estimated by GeSeq were initially approximate, and were adjusted for start and stop positions by comparing expected feature lengths against the references in the annotated genomes listed above. Several genes were omitted from the annotations output by GeSeq, presumably for lack of homology with the references provided in the program and were manually added by searching for them using BlastX searches.

To investigate the identity of the mitochondrial cytochrome c oxidase gene in *C. clypeus*, we aligned genomic reads to the assembled mitochondrion, as well as two putative locations in the nuclear contigs of the assembly, Nodes 86 and 50. These alignments were visualized in Integrative Genomics Viewer v. 2.19.6 (Robinson et al., 2023).

## Results

### Morphological features

**Figure 2:**
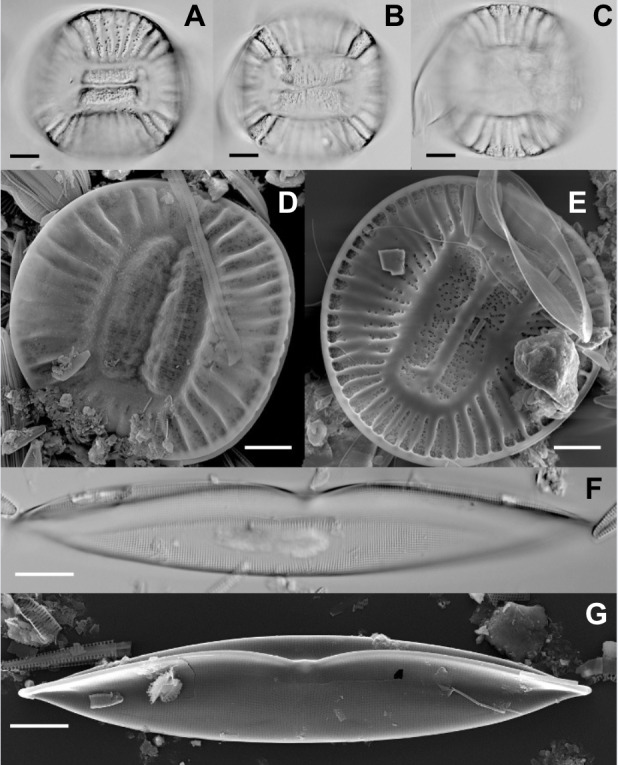
SEM photos of *Campylodiscus clypeus* and *Plagiotropis lepidoptera*. (A–C) Light micrographs of *Campylodiscus clypeus*. Scale bars = 10 µm. (D–E) Scanning electron micrographs of *C. clypeus* (D: Valve exterior; E: Valve interior). Scale bars = 10 µm. (F) Light micrograph of *Plagiotropis lepidoptera*. Scale bar = 10 µm. (G) Scanning electron micrograph of *P. lepidoptera* (Valve exterior). Scale bar = 10 µm.

*C. clypeus* (Ehrenberg) Ehrenberg ex Kützing has angled but nearly circular valves in valve view (Figs. 2A–2C). Valves were 60–84 µm to the long axis, 56–80 µm along the shorter axis. The valves are bent, giving a saddle-shape in girdle view. A slightly raised keel is positioned along the periphery of the valve (Fig. 2D). Valves of this species have an unornamented area that is rectangular in shape in the center of the valve (Fig. 2E). No other *C. clypeus* species were present in the sample from which cells of *C. clypeus* were isolated.

*P. lepidoptera* has linear-elliptical valves with slightly extended apices (Figs. 2F–2G). Valves were 109–140 µm long, 14–17 µm wide. The raphe is within a keel that is elevated above the valve face, except at the valve center. There is a distinct alignment of the areolae longitudinally and transversely. Valves are undulate about the longitudinal axis. This distinctive taxon was the only species of the genus in the sample from which cells were isolated. This may be an undescribed species closely related to *P. lepidoptera* var. *proboscidea* (Cleve) Reimer in Patrick & Reimer (1975) but differing by lacking the asymmetry of the central area illustrated by Reimer (in Patrick & Reimer, 1975).

### Extraction, amplification and sequencing results

Post-amplification, the Qubit readings indicated DNA concentrations of 6.77 ng/µl for *C. clypeus* (total yield was 0.339 µg from 50 µl) and 5.56/7.50 ng/µl for *P. lepidoptera* (total yields were 0.278 µg and 0.375 µg from 50 µl, respectively), confirming successful extraction of genomic DNA and whole genome amplification. These values are within the acceptable range for effective sequencing preparation.

Demultiplexing, read trimming, and quality checks were conducted by the sequencing company using DRAGEN v4.2.7, the onboard analysis software integrated with the NextSeq 2000 platform. A total of 6,252,870 and 8,191,426 million reads were generated for *C. clypeus* and *P. lepidoptera*, respectively, with an average length of 150-bp paired end reads. For *P. lepidoptera*, the same DNA preparation was also used for hybrid sequencing, which generated 6,958,460 short Illumina reads and 256,554 Nanopore long reads (average read length = 2548.9, as reported by Nanoplot).

### Genome assembly

De novo assembly of the metagenomic sequencing data using metaSPAdes resulted in 15,981 contigs (total assembly length = 16,242,939 bp, N50 = 936 bp) for *C. clypeus* and 20,271 contigs (total assembly length = 15,947,516 bp, N50 = 760 bp) for *P. lepidoptera*. To incorporate the *P. lepidoptera* long reads data, a hybrid assembly using another batch of Illumina reads and a set of Nanopore reads was used in metaSPAdes. Hybrid assembly resulted in a total assembly length of 12,630,728 bp and N50 value of 1438 bp.

**Figure 3:**
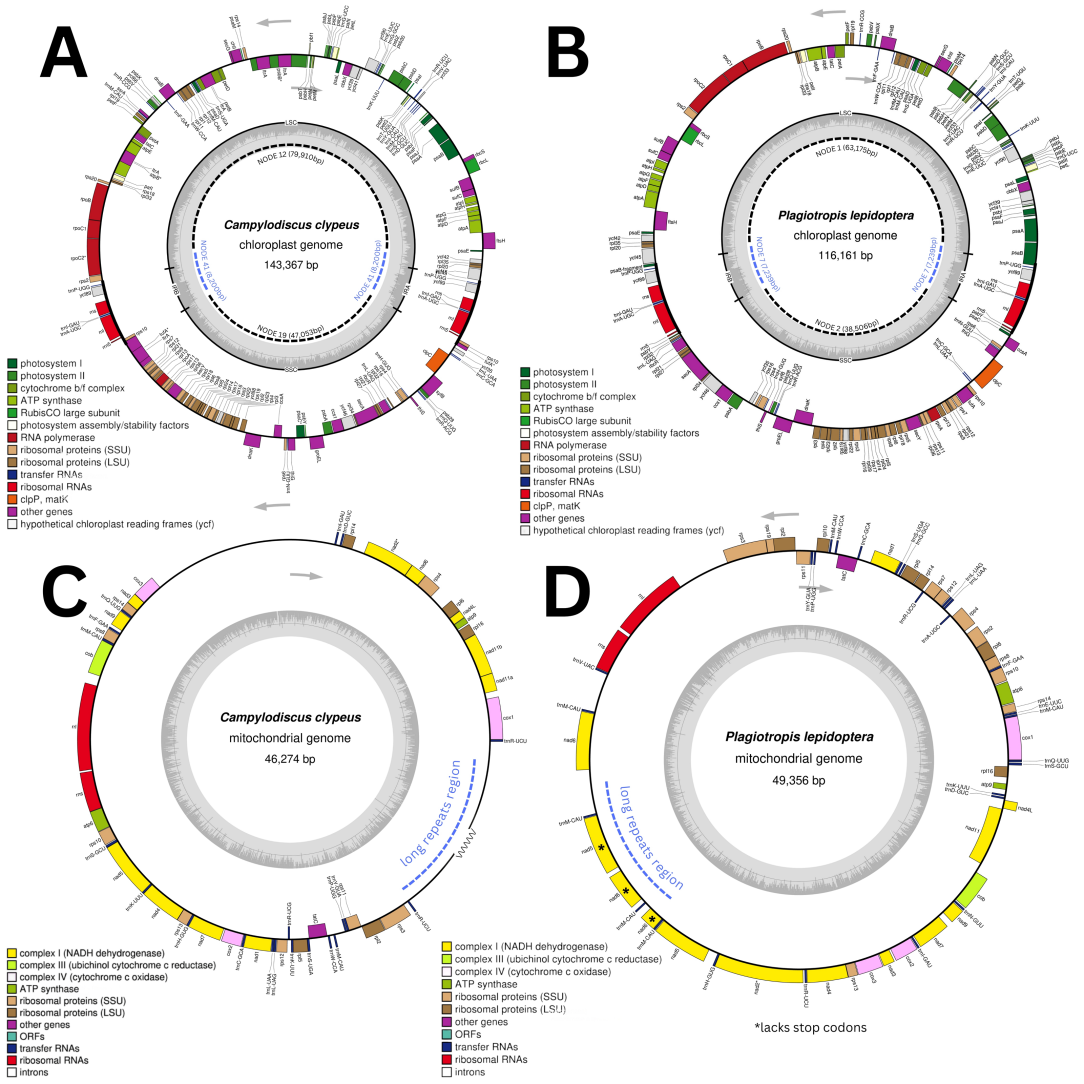
Organellar genome maps of *Campylodiscus* and *Plagiotropis*. (A) Chloroplast and (C) mitochondrial genomes of *Campylodiscus clypeus* (B) Chloroplast and (D) mitochondrial genomes of *Plagiotropis lepidoptera*. The genomes in panels A, B, and D are complete, whereas C represents the complete single-copy portion of the genome, with an unresolved repetitive region represented as a jagged line at the bottom right.

Based on BlastN similarity to known diatom plastomes, the chloroplast assemblies resulted in three contigs each for *C. clypeus* (Coverage/depth = 302, 406, 1,133) and *P. lepidoptera* (Coverage/depth = 102, 111, 259). After duplicating the inverted repeat regions and adding 4 bp in *C. clypeus* and two bp in *P. lepidoptera* during the tiling process, we obtained final genome lengths of 143,367 bp (Fig. 3A) and 116,161 bp (Fig. 3B), respectively (Figs. S1–S6). The chloroplast genomes were circular with a quadripartite structure consisting of two inverted repeat (IRa and IRb) regions positioned between a large single copy (LSC) and a small single copy (SSC) region. De novo assembly often produces organellar genomes in multiple fragments due to repetitive regions, missing sequences, or—in the case of plastomes—the assembler’s difficulty in correctly placing and recognizing the two inverted repeat regions between the LSC and SSC. As a result, it is uncommon for the assembler to reconstruct the entire genome as a single contig, making it necessary to manually stitch the contigs together to form one complete circular DNA molecule. Both genomes were deposited in NCBI GenBank with accession numbers PV231884 and PV231885, respectively.

Given the limited available data for the genus *Plagiotropis*, its identity was further validated by conducting a BlastN of the annotated genes against the NCBI database. The 1,473 bp *rbcL* gene exhibited a 96.00% identity (Query cover = 76%, E-value = 0.0), while the 1,416 bp *psbC* gene showed a 94.92% identity (Query cover = 74%, E-value = 0.0) when compared to *Plagiotropis* sp. strain PR5 cf staurotropidB1 (MH064128 and MH064034, respectively). Both of these hits were the closest matches to any known taxa in the NCBI database.

Mitochondrial genomes were also circular but were smaller in size compared to chloroplast genomes. Both species have a single contig for their mitogenome assemblies, with *C. clypeus* (Fig. 3C) having an approximate length of 46,274 bp and *P. lepidoptera* with 40,104 bp. A repetitive region in the *P. lepidoptera* mitogenome posed a challenge to close the genome. This obstacle was resolved through hybrid assembly, which fully resolved a repetitive mitogenome contig that we were able to join with the single-copy portion of the *P. lepidoptera* mitogenome, resulting in a final length of 49,356 bp (Fig. 3D). Unfortunately for *C. clypeus*, no more viable cells were found in the collection bags. As a result, long-read sequencing could not be performed to resolve the presumed repetitive region. Therefore, we were not able to fully resolve the repetitive region in this mitogenome, and we resorted to filling this region with ambiguous bases. The length of the mitogenome is therefore estimated to be at least 46,274 bp, although the repetitive region likely leaves the genome larger than our approximation. The mitogenomes for *P. lepidoptera* and *C. clypeus* were deposited onto GenBank with the accessions PV266140 and PV266141, respectively.

Although the sequence data for nuclear genomes obtained from our metagenomic assemblies were limited, we were able to identify nuclear gene sequences. The nuclear ribosomal complex containing 18S rRNA, ITS1, 5.8S rRNA, ITS2, and 28S rRNA was recovered in both strains, a region commonly used in phylogenetic studies. Using BlastN, the 4,817 bp contig containing five genes from *C. clypeus* (Coverage = 2.1x) showed 98.52% sequence identity to the query *C. clypeus* isolate LG3.CC (KX120700.1) with 100% query coverage and *E*-value of 0.0. Similarly, the 5,231 bp five-gene contig from *P. lepidoptera* (Coverage = 9.1x) had 93.13% identity to the query *Plagiotropis* sp. isolate IFR16-141 (MG587955.1), with 64% query coverage and an *E*-value of 0.0. The nuclear rRNA/ITS sequences were deposited in NCBI GenBank under the accession numbers PV247950 for *C. clypeus* and PV243986 for *P. lepidoptera*.

### Annotation and genome features

Annotation of the assembled genomes identified protein-coding regions, rRNAs, tRNAs, ORFs, and pseudogenes. The organellar genome features of the two diatoms are summarized in Table 1, with detailed gene content inventories presented in Table 2.

**Table 1:** Summary of basic features of *Campylodiscus* and *Plagiotropis* organellar genomes.

	***Campylodiscus clypeus***	***Plagiotropis*** ***lepidoptera***
**CHLOROPLAST**		
LSC (bp)	79,912	63,175
SSC (bp)	47,055	38,506
IR (bp)	8,200	7,240
Total size (bp)	143,367	116,161
No. of CDS	128	128
No. of rRNAs^*^	3	3
No. of tRNAs	31	30
No. of tmRNA	1	1
**MITOCHONDRION**		
Total size (bp)	>46,274^**^	49,356
No. of CDS	30	37^†^
No. of rRNAs	2	2
No. of tRNAs	17	22

**Notes.**

* One copy of each rRNA gene is present in the Inverted Repeat of the chloroplast genome.

** We presume the existence of a long repetitive region that we were unable to resolve with short reads. This number is a lower-bound for the genome length.

† Some genes (*nad5* and *nad6*) have additional copies that lack stop codons.

**Table 2:** Annotated genes from the plastomes of *Campylodiscus* and *Plagiotropis*. Number of genes per function are represented within parentheses.

	***Campylodiscus clypeus***	***Plagiotropis*** ***lepidoptera***
**Protein-coding genes:**		
Carbon assimilation and metabolism (4)	*rbcL, rbcS, thiG, thiS*	*rbcL, rbcS, thiG, thiS*
Electron transport and ATP synthesis (18)	*atpA, atpB, atpD, atpE, atpF, atpG, atpH, atpI, ccs1, ccsA, petA, petB, petD, petF, petG, petL, petM, petN*	*atpA, atpB, atpD, atpE, atpF, atpG, atpH, atpI, ccs1, ccsA, petA, petB, petD, petF, petG, petL, petM, petN*
Photosystem I (10)	*psaA, psaB, psaC, psaD, psaE, psaF, psaI, psaJ, psaL, psaM*	*psaA, psaB, psaC, psaD, psaE, psaF, psaI, psaJ, psaL, psaM*
Photosystem II (18)	*psbA, psbB, psbC, psbD, psbE, psbF, psbH, psbI, psbJ, psbK, psbL, psbT, psbV, psbX, psbY, psbZ, psb28, psb30*	*psbA, psbB, psbC, psbD, psbE, psbF, psbH, psbI, psbJ, psbK, psbL, psbT, psbV, psbX, psbY, psbZ, psb28, psb30*
Photosystem assembly/ stability factors (3)	*pafI, pafII, pbf1*	*pafI, pafII, pbf1*
Fe-S assembly (2)	*sufB, sufC*	*sufB, sufC*
Antioxidase and proteolysis (2)	*clpC, ftsH*	*clpC, ftsH*
Light harvesting and *chl* biosynthesis (1)	*chlI*	*chlI*
Signal transduction (2)	*cbbX, rbcR*	*cbbX, rbcR*
Protein import (4)	*secA, secG, secY, tatC*	*secA, secG, secY, tatC*
Chaperones (2)	*dnaK, groEL*	*dnaK, groEL*
Transcription and translation (49, 50)	*rpl1, rpl2, rpl3, rpl4, rpl5, rpl6,pl11, rpl12, rpl13, rpl14, rpl16, rpl18, rpl19, rpl20, rpl21, rpl22, rpl23, rpl24, rpl27, rpl31, rpl32, rpl33, rpl34, rpl35, rpl36, rps2, rps3, rps4, rps5, rps6, rps7, rps8, rps9, rps10, rps11, rps12, rps13, rps14, rps16, rps17, rps18, rps19, rps20, dnaB, rpoA, rpoB, rpoC1, rpoC2, syfB, tufA*	rpl1, rpl2, rpl3, rpl4, rpl5, rpl6, rpl11, rpl12, rpl13, rpl14, rpl16, rpl18, rpl19, rpl20, rpl21, rpl22, rpl23, rpl24, rpl27, rpl29, rpl31, rpl32, rpl33, rpl34, rpl35, rpl36, rps2, rps3, rps4, rps5, rps6, rps7, rps8, rps9, rps10, rps11, rps12, rps13, rps14, rps16, rps17, rps18, rps19, rps20, dnaB, rpoA, rpoB, rpoC1, rpoC2, syfB^*^, tufA
**Hypothetical reading frames** (11, 10)	ycf33, ycf35, ycf39, ycf41, ycf42^*^, ycf45, ycf46, ycf66, ycf88, ycf89, ycf90	*ycf33, ycf35, ycf39, ycf41, ycf42, ycf45, ycf46, ycf88, ycf89, ycf90*
**Ribosomal RNA genes:** rRNAs (3)	*rnl, rns, rrn5*	*rnl, rns, rrn5*
**Transfer RNA genes:** tRNAs (27)	*trnA(UGC), trnC(GCA), trnD(GUC), trnE(UUC), trnF(GAA), trnfM(CAU), trnG(GCC), trnG(UCC), trnH(GUG), trnI(CAU), trnI(GAU), trnK(UUU), trnL(UAG), trnM(CAU), trnN(GUU), trnP(GGG), trnP(UGG), trnQ(UUG), trnR(ACG), trnR(CCG), trnR(UCU), trnS(GCU), trnS(UGA), trnT(UGU), trnV(UAC), trnW(CCA), trnY(GUA)*	*trnA(UGC), trnC(GCA),* *trnD(GUC), trnE(UUC),* *trnF(GAA), trnfM(CAU),* *trnG(GCC), trnG(UCC),* *trnH(GUG), trnI(CAU),* *trnI(GAU), trnK(UUU),* *trnL(UAA), trnL(UAG),* *trnM(CAU), trnN(GUU),* *trnP(UGG),trnQ(UUG),* *trnR(ACG), trnR(CCG),* *trnR(UCU),trnS(GCU),* *trnS(UGA), trnT(UGU),* *trnV(UAC), trnW(CCA),* *trnY(GUA)*

**Notes.**

* Pseudogenized gene.

The *C. clypeus* plastome revealed the expected, quadripartite circular genome structure spanning 143,367 bp and containing 128 protein-coding genes, 3 rRNAs (in duplicate, contained within the inverted repeats), 31 tRNAs, and 1 tmRNA. Complete gene sets were present, but *rpl29* gene was not found in the *C. clypeus* plastome. The nearly complete mitogenome of *C. clypeus* spans approximately 46,274 bp and contains 30 protein-coding genes, 2 rRNAs, and 17 tRNAs.

The circular genome of the *P. lepidoptera* plastome revealed a size of 116,161 bp and containing 128 protein-coding genes, 3 rRNAs, 30 tRNAs, and 1 tmRNA. Core gene sets were all present, except for *ycf66*, which was not found, and *syfb* which was pseudogenized in the plastome. The complete mitogenome of *P. lepidoptera* is 49,356 bp in length and comprises 37 protein-coding genes, 2 rRNAs, and 22 tRNAs. For the *nad5* and *nad6* genes, there is one full-length copy and additional shorter copies that lack stop codons (Table 3).

**Table 3:** Annotated genes in the mitogenomes of *Campylodiscus* and *Plagiotropis*. Number of genes per function are represented within brackets.

	***Campylodiscus clypeus***	***Plagiotropis*** ***lepidoptera***
**Protein-coding genes:**		
Complex I (NADH dehydrogenase) (10, 13)	*nad1, nad2, nad3, nad4, nad4L, nad5, nad6, nad7, nad9, nad11*	nad1, nad2, nad3, nad4, nad4L, nad5(x2)^*^, nad6(x3)^*^, nad7, nad9, nad11
Complex II (Succinate dehydrogenase) (0)	None	None
Complex III (Ubichinol Cytochrome-c reductase) (1)	*cob*	*cob*
Complex IV (Cytochrome-c oxidase) (3)	*cox1, cox2, cox3*	*cox1, cox2, cox3*
Protein Import (1, 1)	*tatC*	*tatC*
ATP synthase (2)	*atp6, atp9*	*atp6, atp9*
RNA polymerase (0)	None	None
Ribosomal proteins (SSU) (8, 11)	*rps3, rps4, rps8, rps10, rps11, rps12, rps13, rps14*	*rps2, rps3, rps4, rps7, rps8, rps10, rps11, rps12, rps13, rps14, rps19*
Ribosomal proteins (LSU) (5, 6)	*rpl2, rpl5, rpl6, rpl14, rpl16*	*rpl2, rpl5, rpl6, rpl10, rpl14, rpl16*
**Ribosomal RNA genes:** rRNAs (2)	*rns, rnl*	*rns, rnl*
**Transfer RNA genes:** tRNAs (17, 22)	*trnC(GCA), trnD(GUC),* *trnF(GAA), trnH(GUG), trnI(GAU), trnK(UUU), trnL(UAA), trnL(UAG), trnM(CAU), trnP(UGG), trnQ(UUG), trnR(UCG), trnR(UCU), trnS(GCU), trnS(UGA), trnW(CCA), trnY(GUA)*	*trnA(UGC), trnC(GCA), trnD(GUC), trnE(UUC),* *trnF(GAA), trnG(GCC),* *trnH(GUG), trnI(GAU),* *trnK(UUU), trnL(UAA),* *trnL(UAG), trnM(CAU,),* *trnN(GUU),trnP(UGG),* *trnQ(UUG), trnR(UCG),* *trnR(UCU), trnS(GCU),* *trnS(UGA), trnV(UAC),* *trnW(CCA),trnY(GUA)*

**Notes.**

* Contains additional copies that lack stop codons.

The mitogenome of *C. clypeus* contained the full coding sequence for cytochrome c oxidase I (*cox1*) but would require two implausibly short introns to maintain the reading frame of the protein–that is, introns that are one and two base pairs, respectively. We ruled out assembly error based on the clean alignment of reads to this region (Fig. S5). The assembly contained two full-length copies of *cox1* (1,896 nt, or 631 aa on Node 86; and 1,536 nt, or 511 aa on Node 50) outside of the mitogenome. These contig assembled at the rough coverage-level of the nuclear genome, 4.1 coverage and 7.2 coverage, *versus* the 2–3 coverage of the nuclear genome. Maps of aligned reads are visualized in Fig. S7 (Node 86) and Fig. S8 (Node 50). The CDS of *cox1* on Node 86 contains a 62.2% identity to the pseudogenized *cox1* in the mitogenome (Fig. S6). For Node 50, the identity was 59.1%. A BlastN search of Node 86 against the NCBI NR database reveals no sequences with greater than 78% similarity, but the results are exclusively Stramenopile taxa, primarily *Phytophthora* and *Pythium*. In Node 50, the best hits are to taxa in the Amoebozoa, specifically *Paramoeba*, with 76% identity.

## Discussion

### Optimization of mcWGA technique

Traditionally, obtaining genomic information from diatoms involves isolating single cells and cultivating them in media until unialgal cultures are established with sufficient growth and cell numbers (Fernandez-Valenzuela et al., 2021). Once adequate cell density is achieved, DNA is extracted using a specialized kit (Barbosa et al., 2016). For researchers seeking gene markers, specific regions are amplified *via* polymerase chain reaction (PCR) using targeted primers. In contrast, for whole-genome analysis, genomic DNA is extracted and subjected to Next-Generation Sequencing (NGS) technologies to provide sequence data that samples the entire genome, rather than specific target regions (Gupta & Gupta, 2020).

In the mcWGA approach we have developed and employed in our study, there is no requirement to establish cultures, as individual cells can be isolated directly from raw environmental samples. This method offers an alternative way of analyzing genetic material from uncultured, nonculturable or rare microorganisms. However, it is essential to isolate fresh and viable cells to ensure the effectiveness of the procedure. To maximize the accuracy and completeness of genomic data, isolated cells must be free from debris, contaminants, or other microorganisms, such as other algae or bacteria. Contaminants can interfere with the extraction and amplification process, leading to skewed results or the incorporation of off-target genomic material. Careful sample preparation, aseptic protocols and precision of isolating single cells are critical to ensuring that the amplified DNA predominantly represents diatom genomes, thereby enhancing the reliability and specificity of downstream analyses (Pensold & Zimmer-Bensch, 2020). However, it is important to note that despite the best efforts to isolate single cells free from debris, sequencing results may still yield contaminant DNA. Careful single cell isolations will ensure that the target cell will be amplified with higher coverage compared to contaminant DNA. Table 4 compares the culture-independent mcWGA approach to the traditional culture-dependent method.

**Table 4:** Comparison between culture-independent mcWGA and traditional approaches for generating organellar genome data from diatoms.

	**mcWGA method**	**Traditional method**
**Culturing requirement**	No need to culture cells; direct cell isolation possible	Requires establishing unialgal cultures through multiple isolation steps
**Number of cells needed**	As few as 1–1,000 cells	Requires ∼1,000 to millions of cells
**DNA extraction**	DNA amplification and extraction integrated into mcWGA kit; no separate extraction needed	Requires dedicated genomic DNA extraction kits and often additional purification steps
**Workflow**	Streamlined and fast: - Isolate cells - Follow mcWGA kit protocol - Check DNA yield/quality - Sequence	Multi-step and time-consuming: - Isolate and maintain cultures - Grow cells - Lyse cells - Extract and possibly purify DNA - Check DNA yield/quality - Sequence
**Time efficiency**	Entire protocol can be completed in ∼12 h	Takes multiple days due to culturing and multiple processing steps
**Cost consideration**	Primarily the cost of the mcWGA kit and basic lab supplies	Cost includes: - Culture media - Flasks/Petri dishes - DNA extraction and purification kits - AGE (Agarose Gel Electrophoresis) materials
**Lab equipment & materials Needed**	Minimal setup: - Glass pipettes/slides - Minicentrifuge - Thermocycler - Qubit - Freezer	More extensive setup: - Same as mcWGA, plus: - Autoclave - Incubator - AGE system - UV gel documentation system
**Contamination risk**	Tedious single-cell isolation can increase contamination risk if not done carefully	Lower contamination risk due to established cultures, but more handling steps can also introduce issues
**Purity of sample**	May include mixed-species DNA if isolation is imperfect	Higher taxonomic purity due to use of unialgal cultures
**Genomic integrity**	Minimizes risk of culture-induced mutations by allowing genomic analysis directly from single cells, avoiding prolonged culturing	Requires culturing over time, which may introduce genomic changes due to adaptation or stress in artificial environments
**Scalability**	Scalable for rare or difficult-to-culture taxa	Less feasible for rare taxa due to culture requirements

The success of mcWGA using the Repli-G Single-Cell kit also depends on minimizing DNA fragmentation during processing as the method relies on MDA. MDA struggles with fragmented DNA because Phi 29 DNA polymerase is optimized for long, continuous templates, making it inefficient with short, discontinuous fragments. Fragmentation disrupts strand displacement, reduces primer binding opportunities, and leads to uneven amplification with significant bias (Tšuiko, 2018). Additionally, Phi 29 polymerase performs poorly on templates with nicks or gaps, further diminishing its ability to amplify fragmented DNA effectively (Dean et al., 2002) and stressing the importance of using fresh samples to maximize chances of success.

During the optimization of the technique, we tested a range of cell inputs, from a minimum of three (3) cells to over 30 cells. However, we did not observe a significant correlation between the number of cells used and DNA concentration (in ng/ul) after extraction and amplification; increasing the cell input did not consistently result in a more complete sequencing output. Additionally, we explored other diatom strains outside the genera *Campylodiscus* and *Plagiotropis*. Interestingly, we observed that larger cells yielded better DNA concentrations and more contigs needed to assemble the organellar genomes. However, further studies are required to draw definitive conclusions regarding the influence of cell number and cell size on genomic output.

The buffers, enzymes, and denaturation steps provided in the kit are designed for cells with typical fluid membranes. Because diatoms possess rigid glass cell walls composed of silica, they are significantly more resistant to common lysis procedures and chemicals (Annunziata et al., 2021). Efficient cell lysis is crucial for the release of DNA, to make sure that it will be accessible to amplification enzymes (Eland, Davenport & Mota, 2012; Yuan, Li & Lin, 2015). Also, it is important to preserve DNA integrity without fragmentation for successful whole genome amplification (Pan et al., 2008). This presents a challenge for diatoms, as most methods for disrupting diatom frustules are abrasive and may shear the DNA (Barba, Grimi & Vorobiev, 2015). This consideration is particularly relevant for future studies involving diatom species with complex cell wall organization and interlocking structures in their girdle bands (Kooistra & Pohl, 2015), which may require preliminary steps to effectively break the cell walls and release the DNA. In the case of *C. clypeus* and *P. lepidoptera* strains we isolated for our study, no additional steps or physical disruption methods were performed to break their frustules.

### Limitations and considerations of the minimal-cell genomic approach

Isolating and pooling a minimal number of cells for DNA extraction and amplification can be an effective strategy for obtaining genomic data from taxa that are unculturable, difficult to maintain, or slow growing in culture. However, this approach should be applied with caution. In cases where cryptic species, strain-level diversity, or morphologically indistinguishable congeners coexist, even a small pool of cells may introduce mixed signals that complicate downstream analyses. The method is most reliable when applied to relatively large, morphologically distinctive taxa that can be confidently identified under light microscopy, and when the focal organisms are sufficiently abundant in raw samples to allow unambiguous isolation. It can also be particularly valuable for rare taxa, for lineages that are very difficult to maintain in culture, or in laboratory settings without complete materials and facilities for culturing. However, for groups with known cryptic diversity or where closely related congeners co-occur, single-cell isolation or establishing clonal cultures remain preferable. Thus, while minimal-cell pooling provides a practical and efficient alternative in certain contexts, its suitability ultimately depends on the biological characteristics of the target taxa and the research environment.

### Organelle genomic data obtained using the mcWGA technique

Both *C. clypeus* and *P. lepidoptera* plastomes are within the size range of all sequenced diatom plastomes to date, as well as the sizes of the LSC, SSC and the IRs, based on all chloroplast sequences deposited in Genbank (National Center for Biotechnology Information (NCBI), 2025a). They are the first species to have a fully sequenced chloroplast genome for their genera. While diatom plastomes are generally conserved, structural rearrangements were evident between the two raphid diatoms, with reorganization in the orientation and positioning of genes within the LSC and SSC regions (Figs. 3A, 3B).

In *P. lepidoptera* plastome, the Phenylalanine-tRNA synthetase *syfB* gene was very fragmented (72 bp). Previous studies also reported that the *syfB* gene has been lost in most *Pseudo-nitzschia* (Jeong & Lee, 2024), *Thalassionema* (Zhang & Chen, 2022) and *Thalassiosira* (Sabir et al., 2014). The *ycf66* gene has also been lost in *Thalassionema* (Zhang & Chen, 2022) and pseudogenized in *Roundia cardiophora* (Yu et al., 2018). Yu et al. (2018) indicated that *syfB* has been lost seven (7) times and pseudogenized in *Coscinodiscus radiatus*. Additionally, it was confirmed that both *syfB* and *ycf66* have not been found in the nucleus (Zhang & Chen, 2022).

*C. clypeus* and *P. lepidoptera* lack Complex II (succinate dehydrogenase) *sdh* genes in their mitochondrial genomes. The absence of Complex II is associated with decreased mitochondrial function as it affects intermediate steps in the respiratory pathway. Although studies discussing the loss or absence of this gene group in diatom mitochondria are limited, similar patterns have been observed in other groups, such as some apicomplexans, including *Cryptosporidium* species. These apicomplexans have very reduced mitochondria, often referred to as mitosomes, with decreased mitochondrial electron transport chain efficiency. It was proposed that homologs of *sdh* genes had already diverged extensively, making them undetectable using common bioinformatic tools, and further research confirmed the complete loss of these genes in some apicomplexans (Maclean et al., 2022). This suggests that *C. clypeus* and *P. lepidoptera* may share a similar evolutionary pattern with organisms that have reduced mitochondrial function. However, not all diatoms have lost the functions of Complex II. In *Phaeodactylum tricornutum* and *Thalassiosira pseudonana,* fully functional copies of *sdh* genes were present in the nuclear genome (Bowler et al., 2008).

The *C. clypeus* mitogenome appears to have a pseudogenized copy of *cox1*. The *C. clypeus cox1* gene is full-length (1,497 nt), but contains seven internal stop codons at 3 locations throughout the CDS that cannot be explained by assembly error and would require impossibly short introns to resolve. A remotely possible solution to these frame shifts involves ribosomal frameshifting, in which the ribosome shifts to the +1 or +2 frame if either a particular aminoacyl-tRNA, or the release factor, is limited (Harger, Meskauskas & Dinman, 2002). Lacking experimental evidence for this scenario, we also consider the possibility that *cox1* is pseudogenized in the mitogenome. One of the full-length *cox1* copies detected in two putative nuclear contigs (Nodes 86 and 50) may represent a functional copy of the gene that has been transferred to the nuclear genome. The sequences have low similarity to the pseudogenized copy in the mitogenome (66.2% and 59.1%, respectively) and there are no good matches on the NCBI NR database, with the best match for Node 86 being Stramenopile sequences in Phytophthora with a far-from-diagnostic 78% sequence identity. The transfer of fragments of the mitochondrial genome to the nuclear genome (called nuclear mitochondrial DNA fragments, or NUMTs) has been extensively documented, including in Stramenopiles (Tyler, 2009). However, given the low sequence identity of this fragment, we cannot rule out that this gene may represent contamination in the library by a related protist. There remains a need to obtain a more complete mitochondrial genome of *C. clypeus* and related diatoms to resolve the true identity of the *cox1* gene (Ehara, Watanabe & Ohama, 2000; Pogoda et al., 2019).

In a study by Oudot-Le-Secq & Green (2011), long repeat structures were identified in the mitochondrial genomes of the diatoms *Phaeodactylum tricornutum*, *Thalassiosira pseudonana*, and *Synedra* species. *P. tricornutum* and *T. pseudonana* diverged about 90 million years ago (Bowler et al., 2008; Vardi et al., 2009), suggesting that the presence of these repeat structures is likely conserved and may represent a synapomorphy within diatoms, although the specific sequence and lengths of the repeats is probably not conserved. This feature across such a significant evolutionary timespan implies an important functional or structural role in diatom mitochondria. These long repeat structures are also present in *C. clypeus* and *P. lepidoptera*. In the *P. lepidoptera* mitogenome, the repeat region contained several fragmented copies of *nad6* genes. Although a functional single copy of *nad6* gene was present in the genome, the repeat region contained an additional four copies of fragmented *nad6* gene sequences. In the *C. clypeus* mitogenome, a partially resolved repeat region was assembled using the short-read libraries, but lacking long reads, we were unable to fully explore the nature of this region. Nevertheless, the nearly complete 46,274 bp of the *C. clypeus* mitochondrial genome that was assembled using only short paired-end reads still contains the complete set of genes typically found in diatom mitogenomes.

Currently, 90 complete mitochondrial genomes of diatoms are deposited in NCBI, most of which were assembled using paired-end Illumina short reads (National Center for Biotechnology Information (NCBI), 2025b). In our initial attempt to circularize the mitogenome of *P. lepidoptera*, we successfully assembled a 40,104 bp closed genome. However, there was a missing 6,170 bp repeat region that could only be resolved using Nanopore long reads. A previous study examined nuclear data revisiting two (2) diatom reference genomes using long-read libraries. Their aim was to improve and confirm the accuracy of the *T. pseudonana* and *P. tricornutum* genomes. Through a comprehensive analysis of genome assemblies generated from Oxford Nanopore long-reads sequencing, they identified more genes, resolved previously ambiguous genomic regions, gained deeper insights into complex structural variations, and re-assessed the repetitive DNA regions in both genomes (Filloramo et al., 2021). This raises the question of whether all supposedly complete mitogenomes in NCBI are really complete or if short-read libraries are insufficient to fully circularize and resolve repetitive regions in diatom mitochondria.

The minimal-cell approach was unable to generate chromosome-level assemblies necessary for obtaining the full complement of single-copy nuclear protein-coding genes. However, the presence of the common, high-copy 5-gene molecular barcode (18S rRNA-ITS1-5.8S rRNA-ITS2-28S rRNA) in the assemblies of both taxa reinforces the practicality and effectiveness of the minimal-cell method. While the approach seems more suited for organelle genomes—which are small, in high copy number, and more tractable (Cole, 2016)—recovering nuclear sequences that are commonly used in diatom phylogenies allows for the incorporation of this technique within the data constraints of existing phylogenetic studies (Alverson & Theriot, 2005; Alverson, 2008; Lim et al., 2018; Ruck et al., 2016; Theriot et al., 2015). Additionally, this specific contig was used to help validate the identity of the strains in this study alongside morphological identification.

### Single-to-few-cell genomics in previous studies

The application of single-cell technology for obtaining complete organelle genomes remains underutilized in diatom research where existing studies primarily focus on gene markers only (Baker & Kemp, 2014; Hamilton, Lefebvre & Bull, 2015; Lepere et al., 2011). Several studies cited below have successfully implemented the Repli-G single-cell kit for diverse applications, using distinct and specialized methods and targeting different types of organisms.

Single-cell approaches have been extensively applied in conjunction with amplicon-based sequencing to obtain specific gene markers. Lang & Kaczmarska (2011) proposed a protocol using single cell diatom isolations to sequence and amplify the *rbcL* and *ITS* genes using several PCR amplifications. Hamilton, Lefebvre & Bull (2015) were able to recover partial sequences for *rbcL*, *18S* rRNA and *psbA* genes from a variety of freshwater diatoms using 35 single cell isolates. Similarly, Davis et al. (2019) utilized this technique to analyze targeted molecular sequences in chytrid fungi. Gollnisch, Ahrén & Rengefors (2024) manually isolated *Gonyostomum semen* cells using custom-made micropipettes for population genetics research, focusing on the *18S* rRNA and *cox1* genes. Similarly, Mlewski et al. (2018) characterized *Rivularia* cyanobacteria using gene barcodes, while Frantal et al. (2022) used molecular barcodes to study the ciliate *Urotricha*. Guo, Sui & Liu (2016) enhanced DNA yields from low-concentration samples by analyzing the *18S* rRNA v9 region and *actin* genes in nine dinoflagellate species and 11 diatoms. Broader applications of single-cell genomics include studies like Maurer-Alcalá et al. (2018), which combined genomics and transcriptomics to explore genome evolution in ciliates, and Gérard et al. (2018), which investigated the population dynamics of Alphaproteobacteria and Cyanobacteria.

Specialized techniques were also applied to further develop single-cell methods. Yoshino et al. (2022) used hydrogel-based cell encapsulation followed by whole-genome amplification to analyze *18S* rRNA gene sequences from eight phytoplankton species, including three diatoms: *Fistulifera solaris*, *Thalassiosira pseudonana*, and *Phaeodactylum tricornutum*. Baumas et al. (2024) introduced an RNA-fixative gel method to study *16S* rRNA in diatom chloroplasts and cyanobacteria specifically targeting *Thalassioseira guillardi*, *Trichodesmium erythraeum*, *Crocosphaera watsonii*, and zooplankton detritus.

The single-cell approach has also yielded high-quality genomic data over the years. Mansor et al. (2015) generated draft genomes of the bacterium *Achromatium*, revealing metabolic diversity. Wideman et al. (2020) isolated 26 mitochondrial genomes of marine heterotrophic flagellates, including 10 unique, complete mitogenomes, using an automated cell sorter. Schön et al. (2021) employed a similar approach to isolate 43 single cells, achieving a complete mitochondrial genome of the plastid-lacking Picozoa, *Picobiliphyte lepidoptera*, while Nakayama et al. (2024) sequenced the 1.94 Mb genome of the cyanobacterial symbiont of the dinoflagellate *Citharistes regius* using a hybrid sequencing approach combining Illumina and Oxford Nanopore technologies.

## Conclusions

Collectively, these studies show the versatility and use of minimal-cell genomics in investigating diverse organisms and addressing various scientific questions. This study demonstrates the application of minimal-cell genomics to reconstruct complete organelle genomes, as well as all commonly used nuclear sequences, from individual cells of the diatoms *C. clypeus* and *P. lepidoptera*. By addressing the challenges associated with studying microscopic organisms, this method provides a comprehensive view of their genetic structure and content. Future studies should explore the applicability of this single-cell genomic approach to diatoms with reduced or absent frustules, as well as those with alternative structural features, to better assess its broader use across the group. We also emphasize that increasing the number of cells, rather than a single isolated cell, increases the likelihood of successful whole genome amplification and improves genomic coverage, making mcWGA a more robust and reliable method than using a single-cell approach. The approach enables novel insights into the evolutionary history and biology of diatoms, emphasizing the potential of minimal-cell genomics to investigate microalgal diversity and functions. These findings contribute to future studies of other hard-to-culture microorganisms, enhancing our understanding of microbial evolution and function. This study also highlights the need to explore alternative methods for automating and efficiently isolating single cells, paving the way for future advancements and research.

## Supplemental Information

10.7717/peerj.20767/supp-1 Supplemental Information 1 mcWGA video tutorial A step-by-step video guide of the cell isolations and minimal-cell whole genome amplification method for diatoms.

10.7717/peerj.20767/supp-2 Supplemental Information 2 Coverage depth plot for the chloroplast genomes of *Campylodiscus clypeus* and *Plagiotropis lepidoptera* based on the alignment of sequencing reads to their respective final genome assemblies

10.7717/peerj.20767/supp-3 Supplemental Information 3 Coverage depth plot for the mitogenome of *Campylodiscus clypeus* and *Plagiotropis lepidoptera* based on the alignment of sequencing reads to their respective final genome assemblies

10.7717/peerj.20767/supp-4 Supplemental Information 4 Illustration of the tiling process used to validate the final plastome assembly of *C. clypeus* Raw sequencing reads are aligned across the junctions between plastome regions (LSC-IRB; IRB-SSC; SSC-IRA; IRA-LSC) to confirm seamless connections and assembly accuracy. Reads shown in blue represent sequences with more than one copy in the genome, highlighting the inverted repeat (IR) regions.

10.7717/peerj.20767/supp-5 Supplemental Information 5 Illustration of the tiling process used to validate the final plastome assembly of *P. lepidoptera* Raw sequencing reads are aligned across the junctions between plastome regions (LSC-IRB; IRB-SSC; SSC-IRA; IRA-LSC) to confirm seamless connections and assembly accuracy. Reads shown in blue represent sequences with more than one copy in the genome, highlighting the inverted repeat (IR) regions.

10.7717/peerj.20767/supp-6 Supplemental Information 6 cox1 regions in the mitogenome of *Campylodiscus clypeus* Illustration of genomic reads aligned to three regions in the coding-region of the putative *cox1* sequence in the mitochondrial genome of *Campylodiscus clypeus*.

10.7717/peerj.20767/supp-7 Supplemental Information 7 BLAST Table of the *Campylodiscus clypeus cox1* CDS against the denovo WGS assembly containing putative nuclear contigs for a nuclear copy of *cox1* A BLAST table performed with the CDS of the mitochondrial cox1 in *Campylodiscus clypeus* queried against the whole-genome assembly, which includes any nuclear contigs that could contain a nuclear-encoded *cox1*. None of the results besides the mitochondrial genome contained a functional CDS of *cox1*.

10.7717/peerj.20767/supp-8 Supplemental Information 8 Node 86 for *cox1* Node 86: A contig occurring at roughly the depth of the assembled nuclear genome (4.1 coverage) contains a full-length, functional copy of *cox1*, indicated with the black box. However, the taxonomic identity of this contig remains uncertain, with the best BLAST hit being a 78% hit to non-diatom Stramenopiles.

10.7717/peerj.20767/supp-9 Supplemental Information 9 Node 50 for *cox1* Node 50: Another putative nuclear contig containing a full-length copy of *cox1*, indicated by the black box. The identity of this contig, as revealed by BLAST is less likely to belong to *C. clypeus*, with the best hit being a 76% identity hit to an Amoebozoa, Paramoeba.

10.7717/peerj.20767/supp-10 Supplemental Information 10 Non-mitochondrial sequences of putative cox1 nuclear contigs The contigs resulting from the BLAST search illustrated in Fig. S5.

## Additional Information and Declarations

### Field Study Permissions

The following information was supplied relating to field study approvals (i.e., approving body and any reference numbers): Collection permit was obtained and approved for Joshua Tree National Park, California.

### Data Availability

The following information was supplied regarding data availability: The video tutorial for the mcWGA technique is available in the Supplementary File and at Figshare: Chang, Aimee Caye; Amaral, Mailor Wellinton Wedig; Keepers, Kyle G.; Kociolek, John Patrick; Li, Jingchun; E. Hamsher, Sarah; et al. (2025). mcWGA. figshare. Media. https://doi.org/10.6084/m9.figshare.29473958.v8
All data are available in NCBI GenBank: PV247950, PV243986, PV231884, PV231885, PV266140 and PV266141.
