# Supplementary material for: An alternative technique for organelle genome recovery in diatoms using culture-independent, minimal-cell whole genome amplification
Source: PeerJ. 2026 Feb 25;14:e20767. doi: 10.7717/peerj.20767 (PMC12949581; doi:10.7717/peerj.20767)
Supplement: Supplemental Information 1 — A step-by-step video guide of the cell isolations and minimal-cell whole genome amplification method for diatoms. [file peerj-14-20767-s001.docx]

**MOVIE S1.** A step-by-step video guide of the cell isolations and minimal-cell whole genome amplification method for diatoms. Uploaded on <https://figshare.com/articles/media/mcWGA_Tutorial_Video_mp4/29473958>

< mcWGA Tutorial Video.mp4>
