# Supplementary material for: An alternative technique for organelle genome recovery in diatoms using culture-independent, minimal-cell whole genome amplification
Source: PeerJ. 2026 Feb 25;14:e20767. doi: 10.7717/peerj.20767 (PMC12949581; doi:10.7717/peerj.20767)

**FIGURE S1**. Coverage depth plot for the chloroplast genomes of *Campylodiscus clypeus* and *Plagiotropis lepidoptera* based on the alignment of sequencing reads to their respective final genome assemblies.


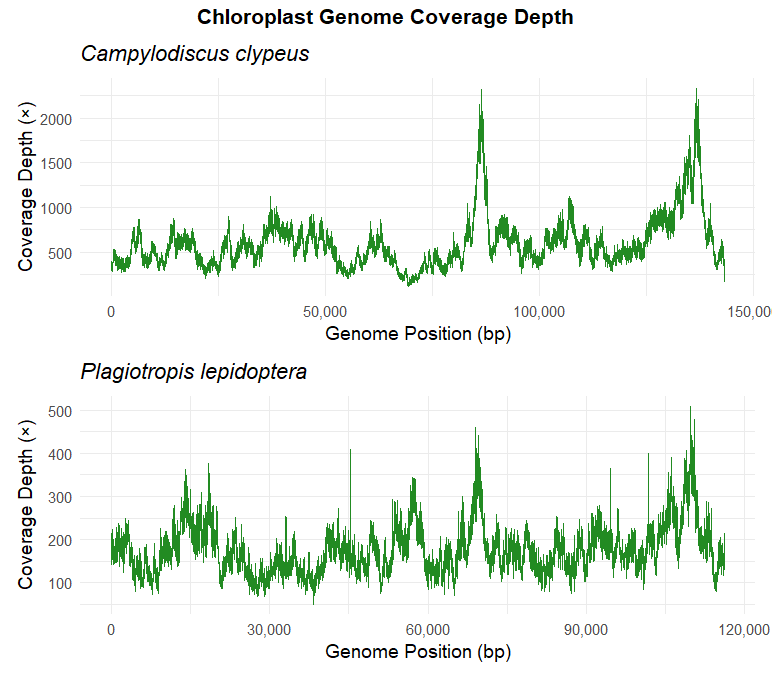

Supplement: Supplemental Information 2 [file peerj-14-20767-s002.docx]
