# Supplementary material for: An alternative technique for organelle genome recovery in diatoms using culture-independent, minimal-cell whole genome amplification
Source: PeerJ. 2026 Feb 25;14:e20767. doi: 10.7717/peerj.20767 (PMC12949581; doi:10.7717/peerj.20767)

**FIGURE S4**.  Illustration of the reads mapped to the final plastome assembly of *P. lepidoptera* using IGV. Raw sequencing reads are aligned across the junctions between plastome regions (LSC-IRB; IRB-SSC; SSC-IRA; IRA-LSC) to confirm seamless connections and assembly accuracy.

**LSC-IRb junction (position 63,177):**


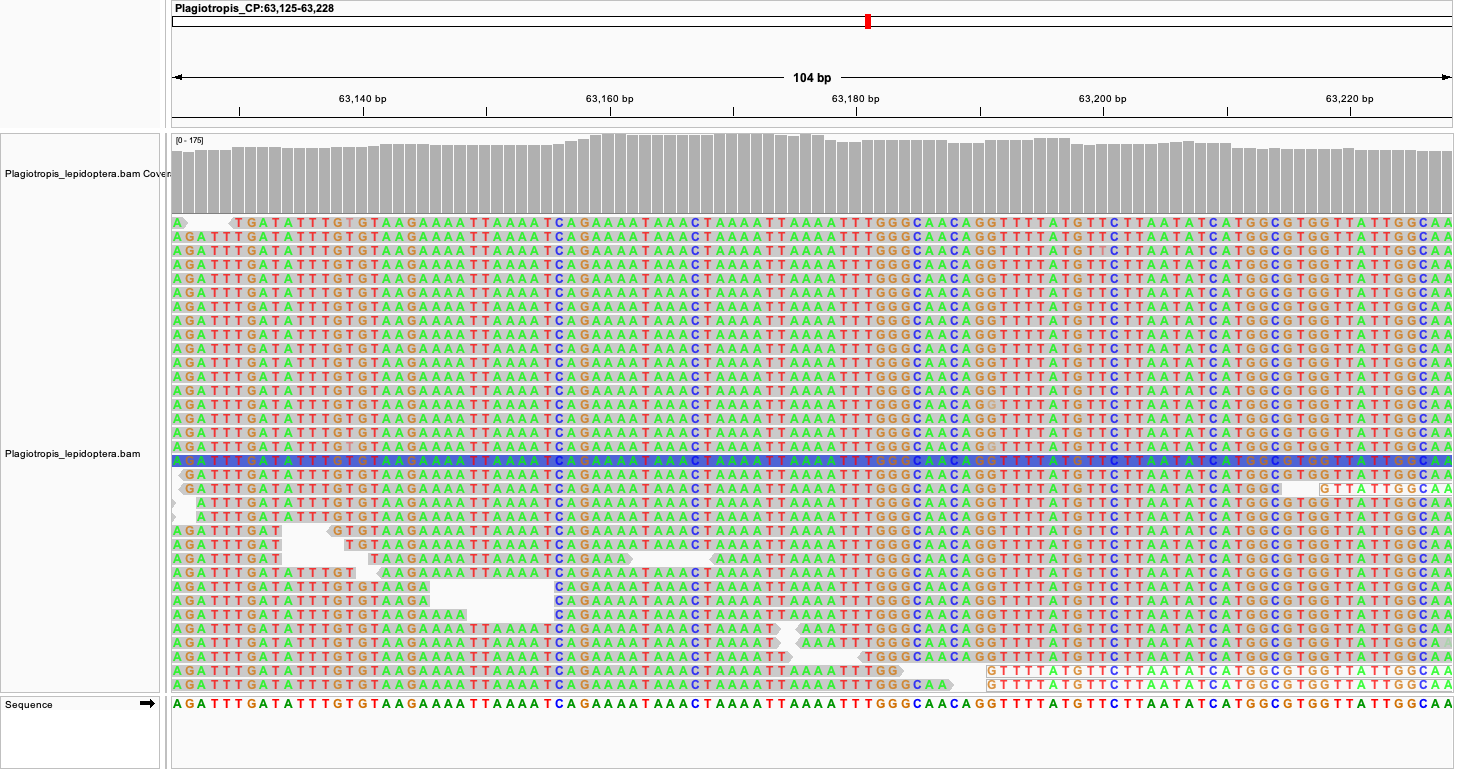


**IRb-SSC junction (position 70,415):**


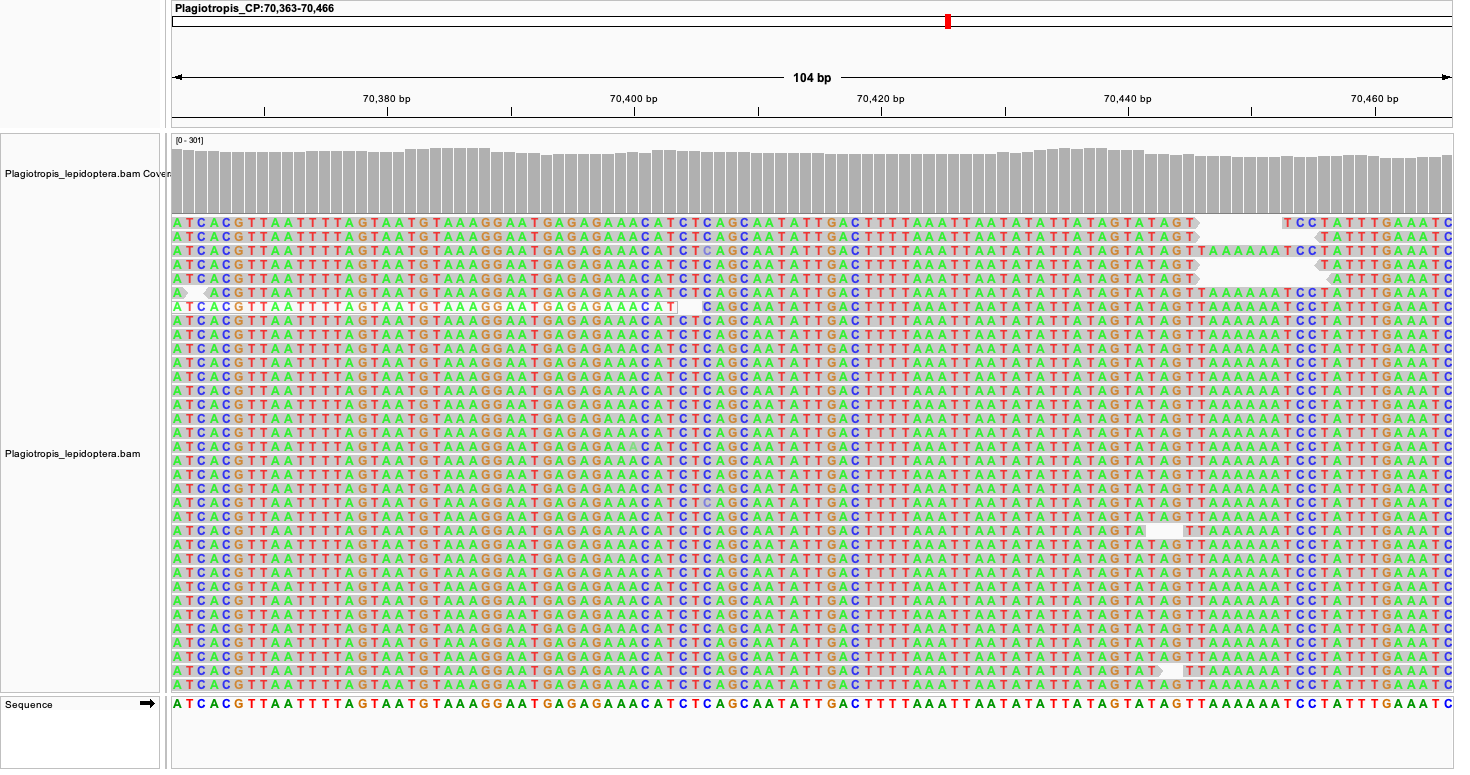


**SSC-IRa junction (position 108,922):**


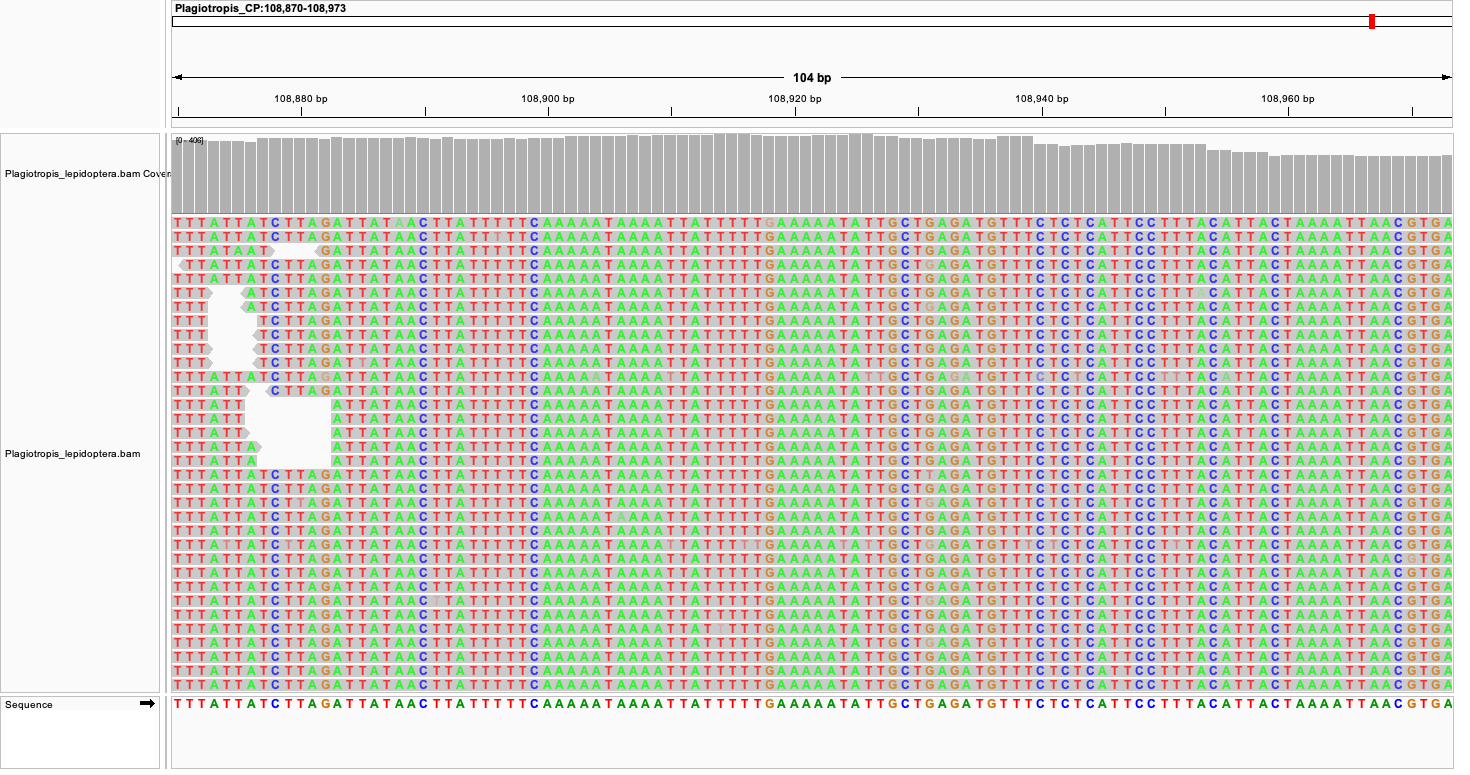


**IRa-LSC junction (position 1):**

**Note: *Input file readjusted to cover starting and end positions.***


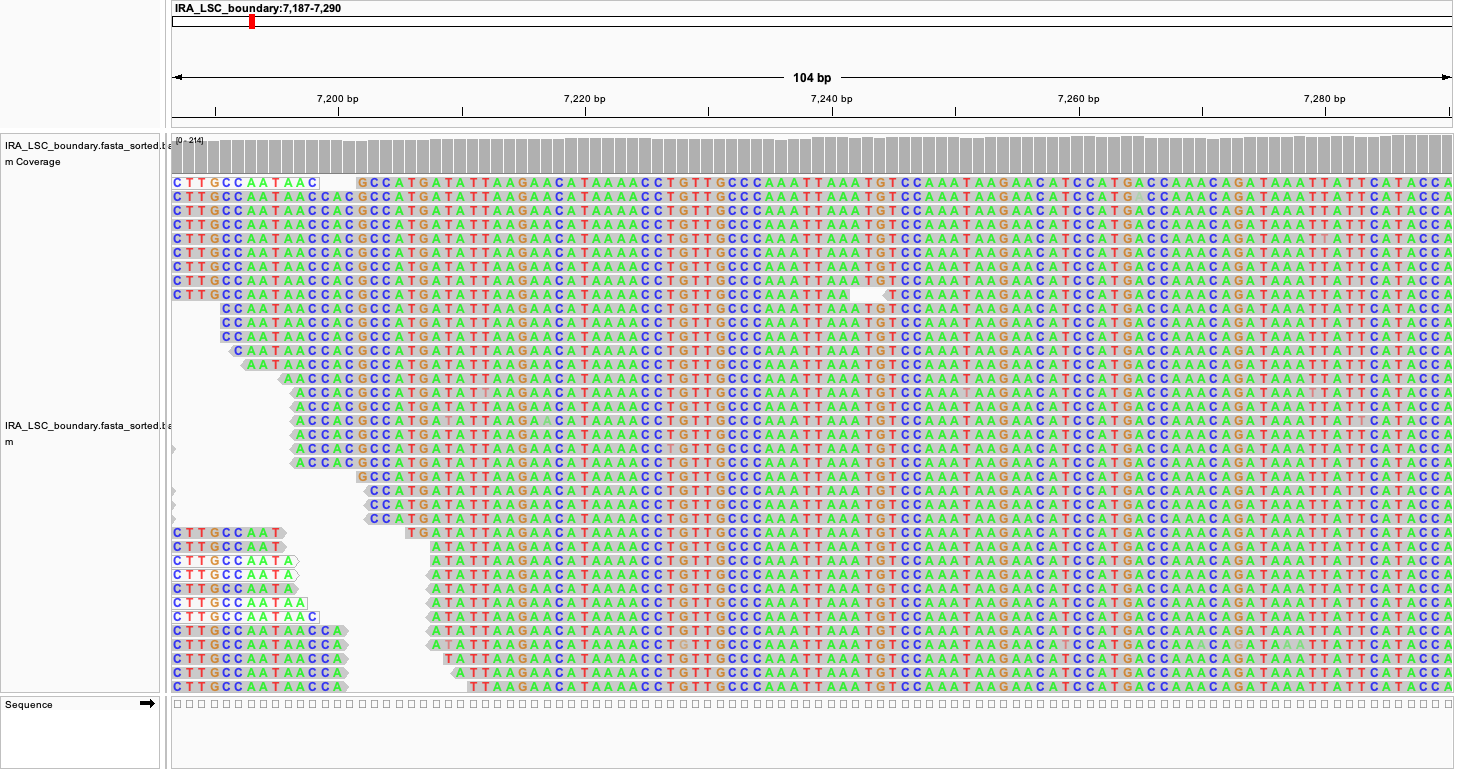

Supplement: Supplemental Information 5 — Raw sequencing reads are aligned across the junctions between plastome regions (LSC-IRB; IRB-SSC; SSC-IRA; IRA-LSC) to confirm seamless connections and assembly accuracy. Reads shown in blue represent sequences with more than one copy in the genome, highlighting the inverted repeat (IR) regions. [file peerj-14-20767-s005.docx]
