# Supplementary material for: An alternative technique for organelle genome recovery in diatoms using culture-independent, minimal-cell whole genome amplification
Source: PeerJ. 2026 Feb 25;14:e20767. doi: 10.7717/peerj.20767 (PMC12949581; doi:10.7717/peerj.20767)

**FIGURE S5.** Visualization in IGV of genomic reads aligned to the region of the *Campylodiscus clypeus* mitogenome containing a putatively non-functional copy of *cox1*. This region contains seven stop codons at the three locations indicated by the red arrows. The introduction of short frame shifts can recover the full coding sequence of the *cox1* gene, but this is mechanistically improbable.


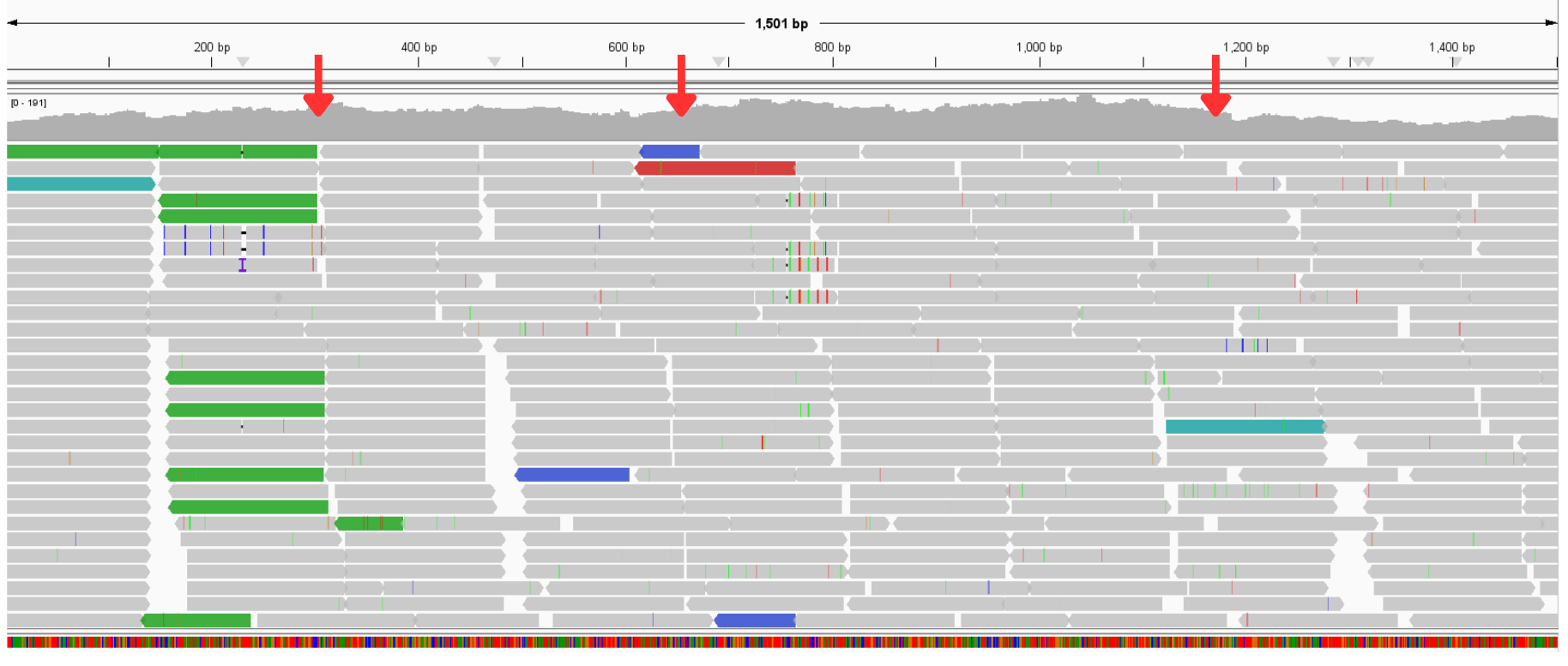

Supplement: Supplemental Information 6 — Illustration of genomic reads aligned to three regions in the coding-region of the putative cox1 sequence in the mitochondrial genome of Campylodiscus clypeus. [file peerj-14-20767-s006.docx]
