# Supplementary material for: An alternative technique for organelle genome recovery in diatoms using culture-independent, minimal-cell whole genome amplification
Source: PeerJ. 2026 Feb 25;14:e20767. doi: 10.7717/peerj.20767 (PMC12949581; doi:10.7717/peerj.20767)
Supplement: Supplemental Information 7 — A BLAST table performed with the CDS of the mitochondrial cox1 in Campylodiscus clypeus queried against the whole-genome assembly, which includes any nuclear contigs that could contain a nuclear-encoded cox1. None of the results besides the mitochondrial genome contained a functional CDS of cox1. [file peerj-14-20767-s007.docx]

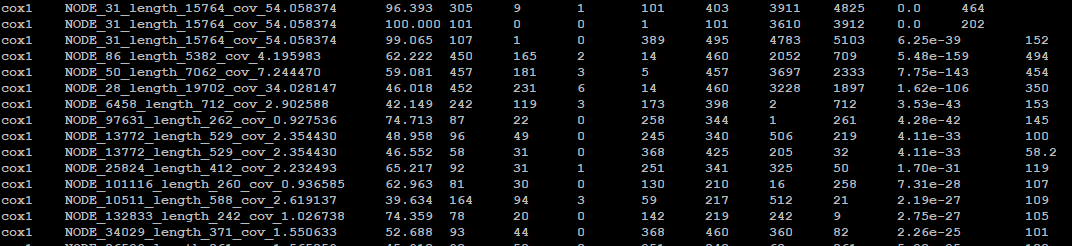
**FIGURE S6.** A BlastN table performed with the CDS of the mitochondrial *cox1* in *Campylodiscus clypeus* queried against the whole-genome assembly, which includes any nuclear contigs that could contain a nuclear-encoded *cox1*. None of the results besides the mitochondrial genome contained a functional CDS of *cox1*.

BLAST Table of the *Campylodiscus clypeus cox1* CDS against the denovo WGS assembly containing putative nuclear contigs for a nuclear copy of *cox1*.
