# Supplementary material for: An alternative technique for organelle genome recovery in diatoms using culture-independent, minimal-cell whole genome amplification
Source: PeerJ. 2026 Feb 25;14:e20767. doi: 10.7717/peerj.20767 (PMC12949581; doi:10.7717/peerj.20767)

**FIGURE S7.** Node 86: A contig occurring at roughly the depth of the assembled nuclear genome (4.1 coverage) contains a full-length, functional copy of *cox1*, indicated with the black box. However, the taxonomic identity of this contig remains uncertain, with the best BlastN hit being a 78% hit to non-diatom Stramenopiles.


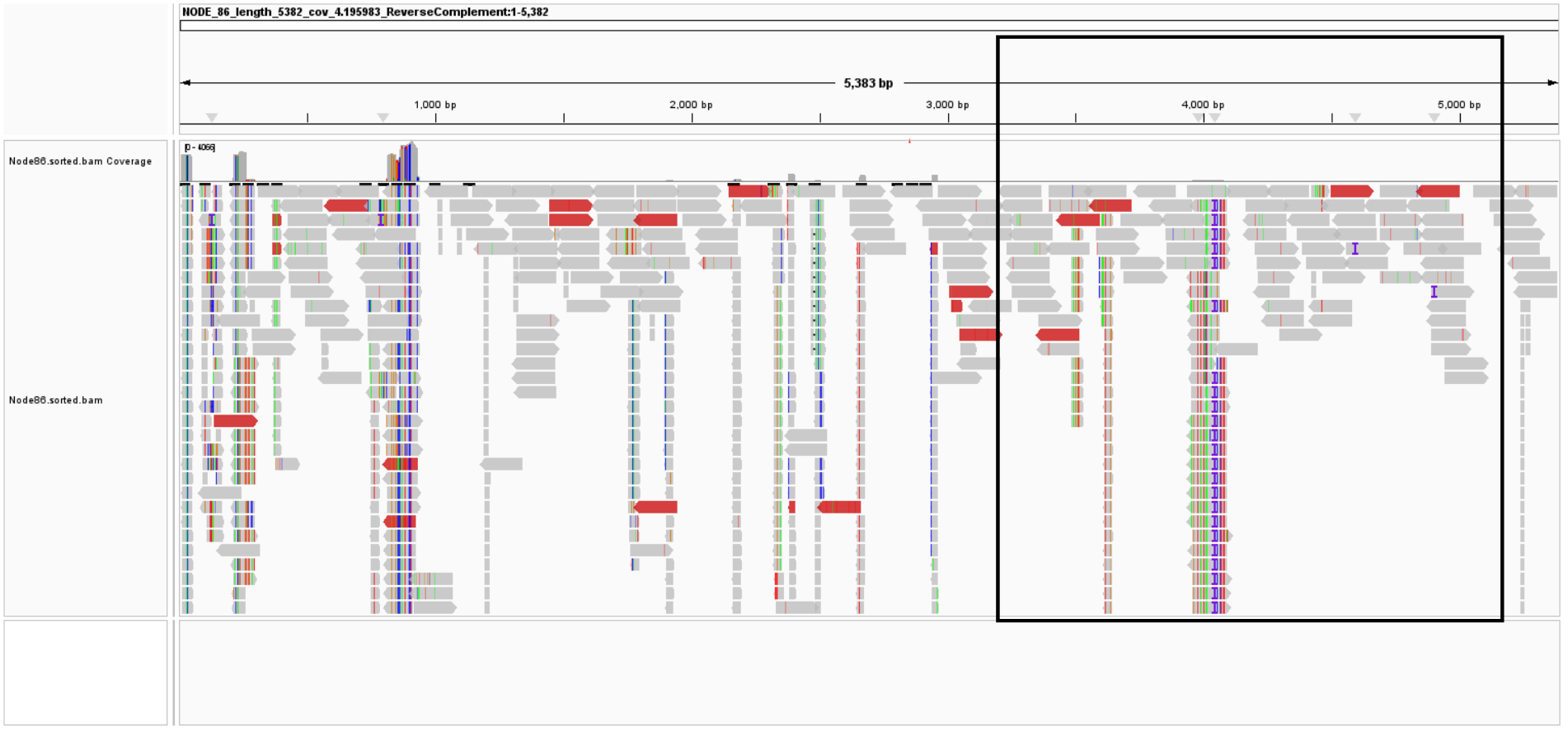

Supplement: Supplemental Information 8 — Node 86: A contig occurring at roughly the depth of the assembled nuclear genome (4.1 coverage) contains a full-length, functional copy of cox1, indicated with the black box. However, the taxonomic identity of this contig remains uncertain, with the best BLAST hit being a 78% hit to non-diatom Stramenopiles. [file peerj-14-20767-s008.docx]
