# Supplementary material for: An alternative technique for organelle genome recovery in diatoms using culture-independent, minimal-cell whole genome amplification
Source: PeerJ. 2026 Feb 25;14:e20767. doi: 10.7717/peerj.20767 (PMC12949581; doi:10.7717/peerj.20767)

**FIGURE S8.** Node 50: Another putative nuclear contig containing a full-length copy of *cox1*, indicated by the black box. The identity of this contig, as revealed by BlastN is less likely to belong to *C. clypeus*, with the best hit being a 76% identity hit to an Amoebozoa, Paramoeba.


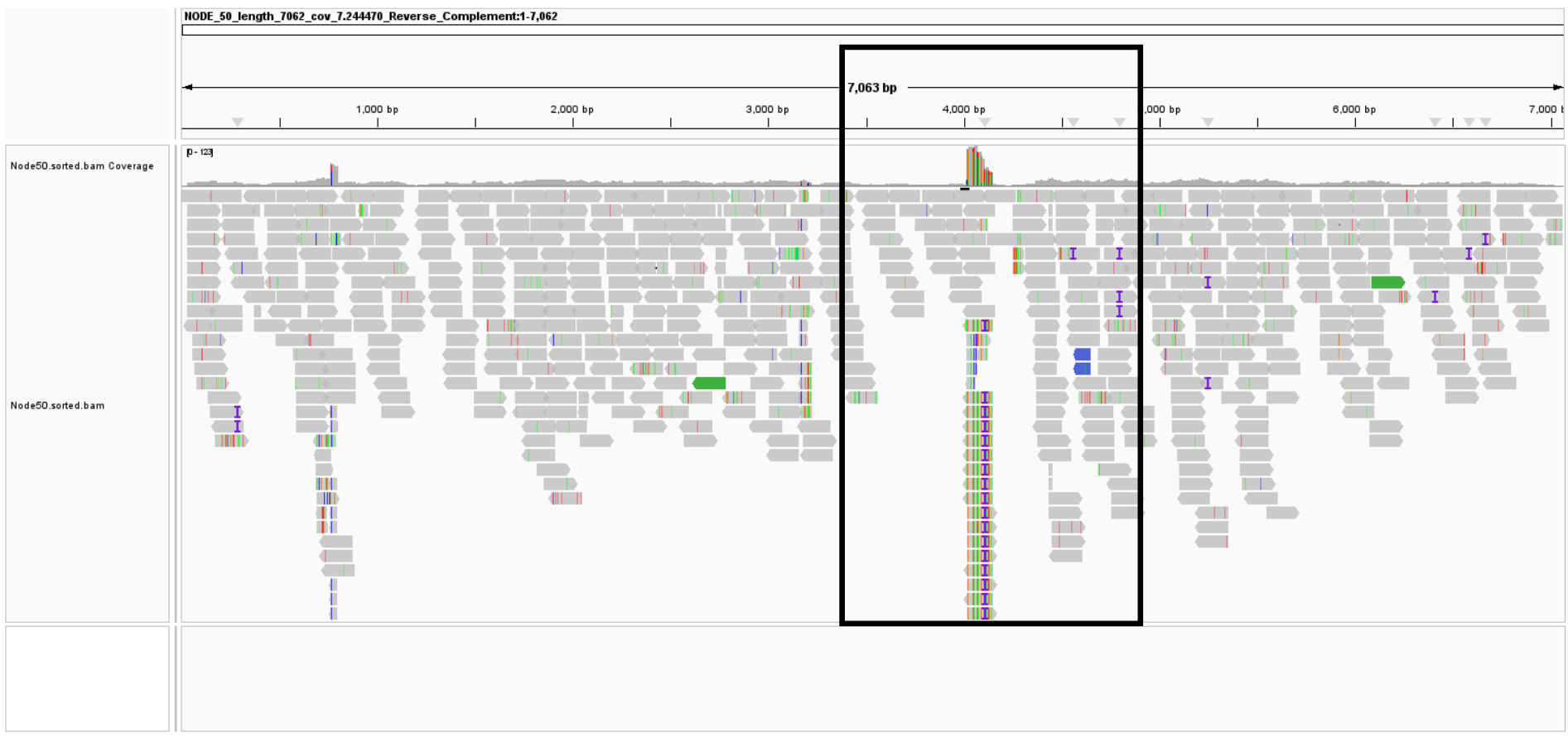

Supplement: Supplemental Information 9 — Node 50: Another putative nuclear contig containing a full-length copy of cox1, indicated by the black box. The identity of this contig, as revealed by BLAST is less likely to belong to C. clypeus, with the best hit being a 76% identity hit to an Amoebozoa, Paramoeba. [file peerj-14-20767-s009.docx]
