# Supplementary material for: An alternative technique for organelle genome recovery in diatoms using culture-independent, minimal-cell whole genome amplification
Source: PeerJ. 2026 Feb 25;14:e20767. doi: 10.7717/peerj.20767 (PMC12949581; doi:10.7717/peerj.20767)
Supplement: Supplemental Information 10 — The contigs resulting from the BLAST search illustrated in Fig. S5. [file peerj-14-20767-s010.docx]

**DATA S1**: The contigs resulting from the BLAST search illustrated in **Supplementary Figure 6**.

Non-mitochondrial sequences of putative *cox1* nuclear contigs

>NODE_50_length_7062_cov_7.244470
GCTCTAACCATTGAGCTAACTATTATACAATTTAAAAAAACATAAAATTATATATATAGGAAGAGCAGGACTCGAACCCACATCAATGGCTCATGAGACCATCAAGTTACCTATTACTCCATCTTCTTTTCCAAATTAATATTATTTATTTCTTAGGTAGAAAATGAGATTCGAACTCATACTACTGAAGTCACAATCCAGCGTGCTAACCATTAACACTATTTCTTCTTCTTTTATAAGTGGTTGGATTCGAACCAACGTTGTATTATACAATAGATTTACAGTCTACCGCTTTAAACCGCTCAGCCACACTTTTTAAATAAACGTTATAATAAATACGCAAAAACAATCAATATACTTTTTATAAATATAAACCACCCTAAAAAAAAAAAAAAATAAAAAAACAAAACACAAAAAATTTTAATTTATAAATTAAAAAAAATAAAAAAACTTTTTACATTAAATATGATGAAAAAAAAGAAAAGAAAAGATAGGTTTTTGTAAAACATTTTTTTAAAAAAAGCATTTTTAAAAAATATAAAATACAAAAAATAAATAAATTTTTATAAAAAAACAGTCTTTAGAGCTATATAAAAACAAGCGTAATTCGTATTTTATTATAAAAACAGAACGCAAATCAATTTTTGCGTTTTTTTGCATTTTTAAAAAATATAAAATACAAATACTTTTAAAACAAAAAAACAAGAAGTTATTCAGGTGAGGGGACTCGAACCCCTAAAAAATAGTTTCTAAGACTAACATGTTTACCAATTTCATCACACCTCTCAAAAAAAAAATTAAAGGATATTAATTAAGTAAATAATATCAAAAAAGCCATCATCAAAGCAAAAAGAGCAATTGCTTCTGTTAAAGCAAATCCAAGAAGAGTATAACTAAACAATTCATTACGTAATTGAGAATTACGAGAATAAGCAATTAAAAAAGCACCGAAAACAACACCAATACCAACTCCAGCACCAGCTAAACCTATAGTTGCAAGACCACCACCAATTTTTTTGGCAACAATTAATAAAATATCTAAATTATTCATTTTTATTTAAAAAAAGATTAAAATATTATATTAAAATTCTTGTTTATATACACACACAAAGAAATTGTTTTAATAAAAAAAAAAATAAAAACATAAAAAATATAATTTTTATGAAAAATTAATCAAGATGAAGGGACTCGAACCCTTACGTAACAAAATTACAATAGATTTTAAGTCTATCATGTCTACCATTCCATCACATCAAAATTAAAAAAAATACAAATGAAGGGACTCGAACCCTTACGTAACAAAATTACAATAGATTTTGAATCTACCATGTCTACCATTCCATCACATTTGTTTTTGCAAATGAGGGGACTCGAACCCCCAACCCCTAACTCGACAGGTTAGTACTTTACCAATTAAGCTACATTTTCAGAATGATAGGACTCGAACCTATATCGTCTGTTTCCAAAACAGATATTTTACCTTTAAACTACATTACAAAATTCCGGTATAGGACTCGAACCTATATTATTGATTTCGAAGATCAATATTTTACCAAATTAAATTAACCAAACAAAAGGTATTAATAGGACTCGAACCTATATCTTAAATTTCGTAGATTTATATTCTTTACCAATTAAACTATAATACCAAAGAACTGCGGAATCGGATTCGAACCGATATTAAAAGATTTGCAATCATTTGTAATTAACCATTATACTATCCGTTTAAAACCATGGTATAGGACTCGAACCTATATCTTGTGATTTAGAAAATCAATATTCTATCCGTTTAAAACTAACCATGCATAAAAAAACAGTCTCGATAGGACTCGAACCTATGTCATTAGTTTTGAAGACTAATATGTTAACCTCTACACTACAAGAAAAAAAGATTATGGTGGGATTCGAACCCACGGTGCATTTTTAATACACGATCCTTTTCAAGAGGATTACTTTAAACCGCTCAGCCACATAAATATTTTCTAGGGGTAGGACTCGAACCCACACGTTTTGCTTTCAAAAAACAACGCCTTACCTTTCAGCCACCCTTTTAATATTTCTCGTAGGACTCGAACCTACATCAGTACTGTTATGAGCAGTATATCTTAACCTTTAGACTAGAGAAATATAAAAAAAAATAAAAATATATTTATACTTTTATTTTATTAACTAAATAAAGTTTAACATAAAATTGAGTAAAAAAAAAATTATAAAGTTTTCAAATAAAAGGTATATAATATCTTTCTCTAAAAGTTCAATATAAAAAAAGACTAAATCAAATCAAACCAACAGTTGTTACTGTACTACCAAAAGAACTTACTGCATTTCAAGTATGATAAGCATCAGGAAAATCAGGAATACGACGAGGCATACCAGCTATACCTAAAAAATGTTGTGGTAAAAAAGTAATATTTACACCTACAAAAATAACTCAAAAAGTTATTTGACCAACAATATCTATATTGTTATAATAACCAGTTATTTTTGGTAATCAATGAAAAAAACCTGTAAAAAGACCAAAAACAGCACCCATAGATAAAACATAATGAAAATGTGCAACAACATAGTAAGTATCATGCATTGCAATATCAATAGAACCTTGTGATAAAACAACACCAGTAACACCACCAGCAGTAAAAAGAAATAAAAAACCTGTTGCAAAATAAAAAGGTGTACGTCATACAATAATACCACCGTAAATAGTTGCTAATCAACTAAAAATTTTAACACCTGTAGGAATAGCAATAATCATTGTACTAGCAGTAAAATAAGCTCTTGTATCAATATCCATACCAACAGTGTACATATGATGTGCTCAAACAATAGAACCAAAAAATGCAATAGAAACCATAGCGTAAACCATACCTTCATATCCAAAAACTGGTTTATTTGTATAAACGCTTAAAACAGTACTAATAATACCAAAAGCAGGAAGTATTAAAACATAAACTTCTGGATGACCAAAAAATCAAAATAAATGTTGATAAAGAATTGGATCACCACCACCAACAGGATCAAAAAAAGTAGTATTAAAATTTCTATCGGTTAAAAGCATTGTAATAGCACCAGCTAAAACTGGTAAAGTTAAAACTAATAAAAATGCAGTTAAAAATACAGATCAAATAAATAAAGGTAAACGATATAAAGTTAATCCTTTTACACGCATATTTATAATTGTTGTCATAAAATTAATTGAACCAGCAATAGATGAAACACCAGATAAATGTAAACTAAAAATTGCTAAATCAACAGACCCCCCAGAGTGCGATGTTATGCTAGAAAGTGGAGGATAAACAGTTCAACCAGTACCAGCACCTGTTTCAATTAAAGCAGATAAAATTAATAAAATAAAAGAAGGAGGAACCATTCAAAAACTTAAGTTATTTAAACGAGGAAAAGCCATATCAGGACTTCCAATCATCAAAGGGATAAATCAATTACCAAAGCCACCTATTAAAGCAGGCATAACCAAAAAAAATACCATGATAAAACCATGTGCTGTAATAACAACATTATAAATTTGATAATCACCACCAAAGAATTGATCACCAGGGGCGCTCAATTCCATTCGCATTAAAATAGAAAAAGTTGTACCAATAACACCAGCGATCATTGAAAAAATTAAGTATAAAGTACCTATGTCTTTATGATTTGTAGAATAGAATCAACGTAAAATATTATTCATTTTAAATTTTTTTTAATTTGCTAAATTTTTTTTAATAATTTGAGAATCTATAGCTATTTTATAGATTATATAAAACAATTTTGCTTTGGGAATCAACTGTATAAAACAGTAAAGAAGAAACCATATCAAAACCTCAAAAGTAGATCATAGTAAATAAAAACAATCAGACGACATCAACAAAATGTCAATATCAAGCTGCTGCTTCAAAACCAAAATGATGCAATCTTGAAAAATGTGATTTTGACATTCTTAAAGCAATAACAGATAAGAAAATTGTTCCTATTATAACGTGGAACCCGTGAAAACCTGTACACATAAAAAAAGTTGAACCATAAATACCATCAGAAATATCAAAAGGAGCAGTTATATATTCAAAAGCTTGAAAAGAAACAAAAAAAGAAGCTAAAAAAATAGTCATAAGCATAAAAAATTCACTTTCACGTTTATCACCTTTTTTTATATATAAGTGTGCTGCTGTTACAGTAGCACCAGATAATAACAAAATCAATGTATTTAAAAAAGGAATATCAAAAGGAGCAAAAACTAAATCGCTCATACCAACAGGAGGTCAACTCAAATTTAAAGCAATTGTTGGTGTTACACTTGATCAAAAAAAACCTCAAAAGAAAGCAACAAAAAACATAACTTCAGAAAAAATAAATAGAAGCATGCTTATAATTAAACTAGCTTGTACTTGTTTGGTATGAAACCCTTCAAAAGTAGCTTCACGTACAACATCTTTAAATCAAGAAAACATTGTTAATAAAACAGCTGTTAACCCAAAATAAAAAATGGCTAAACCAAAGTAATATCCTTGAAAAAAAGTTACTAAACCTAAAGGCATAGATAAACAATTTAAAGCTAAAAGCATAGGTCAAGGACTAATATTTACAATATGTCAGAAATGATAATGTTTAAGAGTAAAATTTTTATTCATTTTTTTTTTTAGAAATTTTTTATATCATTTGTTAAAGTTAATAATAAATTTGATGAAACATAATTTTTATATTTTTCATCATCTAAAATAAAATCTAAATCTTTTCAATTAAAAACAGCATTTATATCATCTGTTTTTATAAAAAAATCATTAATCAAAAAATTTAAAAGATCACTTGAGGCATTAGCACAATAAATTAAATAATCATTTTTACTAACACCATAAATTACTATAGGCATAAAACCATGTTTAACACCACATAATTCACTACATTGACCATAAAAACGTCCTATTCGATTTATAAAGCACATAACTTCATTTAAACGACCAGGAAGGGAATCAACTTTAACACCCAAAGAAGGTATAGCTCAAGAGTGTATAACATCACTTGCAGTAACATAAACTTTAATATAAGTATTTACAGGTATAACTAAAGGATTAGTAACATCCAAAAGACGTAATTCTAAAGGTTTTAAATCTTCTGTTTCTAACATATAGCTATCAAACATAACTTTTATATTTTTGAATGAATAGCTTTCTGAAAATAAAGGTAATGCTTTTAATCTTGTTACACACTCATAGGATCAATATCATTGATGACCAACGACTTTAACAGAAATATTACTATCCATAAAAGTTTCAAAACCATATAATAATGTAAAAGAAGGTAAAGCAATAAAAAGTAGAATAAAACTAGGAATTAAAGTTCATATAACTTCAATAGCAGTATTGTGCGATAAATCAACACTATATAAACTTTTAATTCTAAGAAAATTTTGTATAGAATTTTTGCTATTTACTGTAAAAGAAGTTAAAATAAAATACATCATAGTAAAAACAAAAAAAAGTACAATAACCAAAAAGAACATAATATAATGGTGTAAATTAATTAAACCAGACATCAAGGGAGAAGCTGGGTCTTGAAAACCTAATTGACTAGGTACTGCATAATCCAAAAAGCTTTTTGTTATTAAAAACATTTTATTATCTTATTTAATTTTTTAAAACAATTCAACTACAAAAAGAATGCATAAAATTTTCCAAAAGTTTAAAGTCAATACCAACTTTATTTGGATGAATAAATACATTCAAGAAAGTAAAAAAAGGTTTTCAAAATGGCTTTAAGCTATCAACAACCATTTTTTTGAAAACAACATCGATACTTTTTAAATCAAAAGAATCAAAAAAACCAGTAGCACCACCAAAAACAGATAAAAAAGATAAATCTAAAGAAAAATTATCAAATCTACGTTGTATAAAAAGTTCAGTTAAACGATAACCTCTATCTAAAAGCTGTTGTGTTATAGGATCAATATCACAACCTAAACTCGCAAAGCTTTCAACTTCTCTAAATTGAGCTAAACCAACTTTTAATGAACCAGCAATTGATTTAATTAAACGAGGCTGTGCAGCACCACCGACACGACTAACAGATAAACCAACGCTAACTGCTGGTCTTATACCACTATAAAATTGTTCAGTATCTAAGAAAATTTGACCATCTGTAATAGAAATAACATTTGTTGGAATATAAGCAGAAACATCACCTTCAAGTGTTTCAATAACAGGTAAACCTGTCTGCGAACCAGCACCTAAATTTTTAGATAATTTAGCTGATCTTTCTAATAAACGAGAATGTAAATAAAAAACATCACCAGGATAAGCTTCACGACCTGGAGGTCTTCTTAATAACAATGACATTTGACGATAAGCAACAGCTTGTTTACTTAAATCATCATAAATAACTAAACAATGTTTTTTATTATCTCTAAAATATTCACCTATTGCAGCACCAACATAAGGAGCCAAATATTGAAGAGCAGCAGCTTCTGAAGCATAAGCACTAACAATAACAGTGTAATTAGAAGCTTGAAATTTTTTTAAAGTTTCAAAAATTCTTGTAGAAGTTGATTTTTTTTGACCAATTGCAACATAAACACAATAAACATTACCATTTTTATTATCTATATTTTTATTGTAATTTAATTGATTAATTATGGTATCAACAGCAATAGATGTTTTACCTGTTTGTCTATCACCAATAATAAGTTCACGTTGACCTCTACCTATTGGTATTAAAGAATCTAAAGCTTTCAAACCAGTTAACATTGGTTCATGAACAGATTGTCTAGCAATAATACCAACTGCTTTTTTTTCAACATTAACAACAATTTCTGCATCAACTTTTACATTAGAATCCAAAGTTTCACCAATTACATTGATAACACGACCCAATAATTTTGAAGAAACACCAACTGTCATTAAATTACCAGTATTTCTAACTTTGACACCTTCTTTTATTAATTTTTCATTACCAAAAATAACGGCGCTTACTTCATTATGAGAAAGATTCAAAGCTAAACCTTTTACACCAGTAGAAAACTCTAAAACTTCACCAGCTTTAGCCTTATCAAGACCACGAATAATTGCAATCCCATCA
>NODE_86_length_5382_cov_4.195983
CAGTTAGTTCATCAAACAAAAATGACCAATTCAAAGCAAATAAATTTAAATTAAACCAAGGGACTAAAATAACATTAGTAGTAACTTGAAGAAATACAACCTCAAAAAATAAAAAACTTGAACATAAAAAAGAAATAAAAATAAAACTTGATGAAAATAAGGAAATTATATAGTTTCCCAAAAATCGCGCTGACAAAAAACAAAATAATGAACTAAACAGGGACAAAAAAATTACTAATAAATACATCACATAGAAAAAAATCTAAACTATCTTTCTTCTTTCCGTCCCCTATGTCTATTATTAGGTCTCCACTCTTCATCTATATCGACGTGCTCTACAATCCGAAATAATGGTCCTCGAACAATATAATATCGAGACGGTAGTGTACGTAATTTAACTAATTTGTAAATAAAAAGATAGGCGACTCCAATAAATGAATTTACATATTTTAAAAAAGGCAAACAAAAATAAAAAATTTTTTCACCGCTAAAGGCAGCAAAATATGTTACAAAATCAGTAGAAAAAGATCTATAATGATAAACAAAATTTGGATATTTATTAAATGATTGTGGAACAAATTTCTTAAACCTTGAATTACCCTTCACAGGTTCCAAAACTTGAACTTGTCTTGACAATGAAACAAAAATAATATAAAAAAACAAAATTACAGTAATAATTGACACAAAAGCACCAAACGATGATACACTATTCCACAACGCATAACCATCAGGATAATCAGGAATACGTCTTGGCATACCAGATAAACCTAAAAAATGCATGGGAAAAAACGTAATATTTACAGATGAAAACATAATAATAAAATGTAATCGGCCTAAAAATTCCGGATAACTAACACCAAAAATTAAAGAAAACCAATAATAAAACGCAGCAAAAAATCCAAAAACTGCCCCCATTGACAAAACATAGTGAAAATGAGCAACGACATAATAAGTATCATGAAATGCTTGGTCTAGTCCAGCATTAGCAAGTATTACACCAGTTATCCCTCCTATAGTAAACAAAAAAATTAACCCAACAGAAAAAAGTAATGGAACACGCATAATTAGCCGACCACCCCATAATGTTGCTAACCAACTAAAAATTTTAATTCCAGTTGGCACTGCAATTATCATTGTTGCCGCCGTAAAATAAGCGCGCGAATCAACATTCATCCCAACAGTATACATATGGTGAGCCCAAACAATAAACCCTAAGAATCCAATTGAAACCATTGCGTAAACCATCCCTAAATAACCAAAAATCAATTTTTGAGAATAACTTACAATTACATGACTAATTGTTCCAAACGCCGGAATAATTAAAATATAAACTTCTGGATGACCAAAAAACCAAAATAAATGTTGATACAAAATAGGGTCACCACCACCAGCAGGATCAAAAAATGTAGTATTAAAATTTCTATCGGTTAATAACATAGTAATTCCACCTGCTAATACCGGCAATGATAATAAAAGTAAAATAGCAGTTATAAATACAGCCCAAACAAATAATGGTAACCTGTACCATGTAATCCCCTTTACGCGCATATTTATTACTGTTGTGATAAAGTTAACCGCACCCAATAAAGATGATATTCCAGCTAAATGTAAGCTAAAGATAGCAAAATCAACAGCGGGGCCAGAATGAGCAGAAATCGATGCTAACGGAGGGTAAACAGTCCATCCAGTACCAGCACCAACTTCTGAAAAAGCAGACATTAATAATAGTAAAAAAGATGGTGGTAATAACCAAAAACTAATATTGTTTAAACGGGGAAATGCCATATCTGGTGCACCTATTAATATTGGAACAATCCAATTACCAAAACCACCAATCATTGCGGGCATTAACATAAAAAAAATCATTATAAAAGCATGCGCAGTTATAATAACATTATATAATTGATAATTACCTCCTAAAACTTGAATACCGGGTTGGGCTAATTCCCAACGAATTAAAACAGATAAAGCAGTACCAATAACACCAGCAAACGACCCAAAAATTAAATACAAAGAACCTATGTCTTTATGATTCGTTGAATAAAGCCACCTAAATGACCAATTTTTGAACCCAACAGATCTAAATTTCGAAAACAAACGATAAGATAATTGTAAAAAAATATTTTTAAGTGTTGAGTAATTATAATTTTTTTTAAATCTCATTTATTATTTATCTTTCTATACTTTAAAATAAACTCATTTAGGAATCAAACCTATTAAAAATATTAATGAGTTTTTCGGAATGGGACTTAAACCCATATTAAAAGATTATATCTTGGTTTTACAACAATTAAAACTATCCGAATTAAAAATGGAGTCAATCGGAATTGAACCGATACCAAATGTATGCAAAACATTTATTCTACCAATTAAACTATAACCCCTCCTTCTATAATCCAGGAGCAGATTCCTCTACTCCTACCTTGTTACGACTTCACCCCAATCATGGAGATTGTTATCATAAAACAGCCTAATTTCTGAACAAAAAAGAGACAAATTTTTTACTTCAAACAATCCGCACTCTCGTGGCGTGACGGGCGGTGTGTACGAGACTCATAACGATATTCACCGTAACAGACTTTTTTACGATTACATGTGATTCCAACTTCATGTTGCTGATTTTCAAACAACAATCCGAACTATGGCAAAAAAAACTTTTACGATTACTTTTTTTTACAAAATTGTTTCGCTTTGTAATTACCATTGTAGCACGTGTGTAGCCCAATTTATAAGGGACATACTGACTTGTCATCATCCTTAAGCTTCCTTAATCGCTTTGAAAACAGTTTAAATAGAACAAAAAAAAAATTTATGCACACTATTTATAAGGGTTGCGTTCGTTTCTGGAACAAACCTAAAATGTCACAA

CACGAACTTACGACAGCCATGCAGCACCTGTGATAAAAATTAAAAATTAAGAAGTTATTTAACATATAAATTTTTTATCGCAAAAATTGGTAAGATTGTCGCGTATTCTCGAATTAAACCACATGCTCCACCACTCGTTTGATTCTCCGCCACTTCCTTTAAGTTTCAACCTTGCGGTCGTACTCCCCAGGTGGAGTGCTTATCGCGTTAACTAAAGCGTTCGTTAAAATAAAATTATAATTAAATATATTTTTTTAAGATAATATTAAACTACTTAAATTTTATTTCAAACTCTTAACACTCAACGGTCTCAGTATAGACTACCAGGGTATCTAATCCTGTTTGCTCCCTATACCTTCACACATAAACGTCAGAATAAAAAAAGTTGTTGCCTTCGCCAAATGATAGTCTAAAATAAATCTCAAAGAATTCCAACTCTAAATTTAAAGTACTACAACTCTCTTTTAAACTCAAAGTAAAACAGTTTCACACATATTAAAAAGTTAAGCTTTTGTTTATGTACAGACTTTTTTTACCGCCTACATGTCCTTTACACCCAGTTATGATGAGTAACACTAATCCCCTCCGTATTACCGCGACTGCTGGCACGAACATTAGCCGGGATTTCTTTTTTGGTTAATATCATTATTTTTTCCAACGAAAGAATTTTACAACACAAATAAACAAATGCTGTCATCATTCACCTAGTATTACTGGATCAAGCTTTCGCTCATTGTCCAATATTCCTCACTGCTGCCTCTAAATCGAGTCAGGGCCGTGTTTCAGTCCCAATGTGGCCGATCATTTTCTCAAACCGACTAAAGATCAAAGGCTAAATATTTTTTAAAAATTTACAACCTAATCTTATACAACTTAACCAGTTAACAATTATAAATTTTTTCATTAGCAAAATTTATTTTTATATTAGAAAAATTATTGAACTAAAAATATTTGATATTTTATATCAAATTAACTTTATATATAGCTGTATATTACTCACCCGTTCGCCACTAAAAAAAGATTTTCGTTCGACTTGCATGTGTTAAGTATACTACTAGCGTTCACTCTGAGCCAGGATAAAACTCATTAACAATAAAATATAATTTATATAAAAATTATACTACTAAAATTAATTCCGAGATGGGAATATTGAGAGTCGAACTCAAAACCAATGGTTTAAAAGACCACTGCTCTACCATTGAGCTATATTCCCAAAGTACAAAACACCTCAGATTGGATTTGAACCAATACATTTTTGACTTAGAAGGTCAACGCTTTCCCAATTTAGCTACTGAGGTTTTTTTAAAAAAAAAAAAAAAATCAAGTTTTAATAAAATATACTAACAAAAAAAAACTACATATTACTATGCGACAATATTTTTGTTAAATTATAGTCTACAATAATTTTTAAAAAATTTTATGAAAATATTTTCCTACGATCAACGTAGTTATATAGTCTATGCGTAGCTTCTTGGCACTGCTTCAAAAAAAAACAACCAAAACACCATAGGCATATTTTTTTCAGTCCTCTCGTACTAAAAAAAAAATTTCAAAATTTTTTGAAATTAGTAACAGATAGGAACCGAACTGTCTCACGACGTTCTGAACCCAACTCACGTACCACTTTAATTGGCGAACAGCCAAACCCTTGGAACCACCTACAGCTCCAGGATGTGATGAGTCGACATCGAGGTGCCAAACAACTTCGTTGATAAGAACTCGCAAAAGTTATTAGCCTGTTATCCCCGGCGTACCTTTTATCCGTTAAGCAATAACCTTTCCATACAGCATTATTGGATCACTATGGTCAACACAATGAAGTATTTCTGATCGACTTGTCTGTCTTACAGTTAAGTAAATTTTTGCCATTACACTCTACAATTAATAAAAATTAATTTGAATTTACCTTATACGCACCTCCGTTACTTTTTTAGAGGCAACCGCCCCAGTTAAACTACCAATCAAGCATATTTCTTTTGTATTATTATAAACCAAAATTAGTTTTTGTGCAATAAAAGAATTCTATTTCACTAGTGTCTCCTTAGTTAATTTGCATTAACAACTCATAAACAACAAATTTATTCTACACAAATAAAAACACAAAAACAATACTAAATTATAGTAAAGGTGCACGGGGACTTTCCGTCTTATTACTAATATTCCGCATCTTCACGGATAATTCAAGTTCGCTAAGCCAATGTTAGAGACAGCGGGGCAGTCGTTACACCATTCATGCAGGTCAGCAATTAACTGACAAGGAATTTCGCTACCTTAGGACCGTCATGGTTACAGCCGCCGTTTACTGAAACTTATATTTTGAACCAATTAAATTCTCAATTTTCATTTACCAGCACCGGGCAGGTGTCAGACCCTATACATCATATTTCTATTTAGCAGAGTCCTGTGTTTTTGTTAAACAGTCGCCACCCCCATTTTTGTGACATCAACAATAAATTGATACTTCTTCTCCCGAAGTTACGAAGACATTTTGCCGAGTTCCTTAAACAATGTTAT
>NODE_6458_length_712_cov_2.902588
GATCAATTACATCACCACCGTGATCAACATGAGAACAAAGGGGATGTCCTTTTCAAGACTTCCACTCACGATTTGGGCGTTCTTCTTCACGGCCATTATCGGAGTACTTTCATTTCCGGTATTATTTGCTGCTGCATTACTATTGATATTTGATAGAAGTATGGGGACAAGCTTCTTCTTGTCAGACATATACATCAACGGAGAAGCACTTGCAAATCAGGGAGGTAGCCCTGTGCTATTTCAACACTTGTTCTGGTTCCTTGGTCATCCGGAGGTGTATATTGTAATCCTGCCATCTTTGGGTATCACTTCTGAAATCATTGCGACCAATTCCCGAAAGCCGATCTTTGGCTACAAGGCGATGGTTGGTTCGATCTTGTTCATCACGATACTTTCATTCGTAGTATGGGCGCATCACATGTTTGTGACCGGAATGAACCCATTTTTAGGTTCCATCTTTACTTTGCTTACTTTGATTATCGCTGTACCAAGTGCGGTGAAGATCTTTAACTACGTGACTACCCTGTGGAAAGGTAATATCATCTTTACGCCAGCGATGTTGTTTTCAATTGGTCTGGTGTCATTCTTCCTTTCAGGAGGTATCACTGGAATCTTCCTTGGAAACTCAGCGGTGGATATTCAATTACTTGATACGTATTTTGTAGTAGCTCACTTTCATCTTGTGATGGGCGCTGCATCTTTCTTCGGTATG
>NODE_10511_length_588_cov_2.619137
CTTCTATCGAAGATAAGCAGCAGGGCAGCGGCAAATAAAACCGGGAATGACAATACGCCAATGATTGCAGTGAAGAAGAACGCCCAAATGGTCAAAGGCAGTCGGGAAAAAGACATACCCTTGGTGCGCATGTTAATAACTGTAGTGATGTAGTTGATACCTCCCAACAAAGAGCTAACAATAAAAAGAGCCATCGCAACCAACCACAGCGTCATTCCCAATCCTGATCCTGACATGGCTTGTGGCAAGGCACTTAATGGTGGATAAATAGTCCATCCTCCACTGGGAGGACCAGTAGAAAGGAAAATAGATGTAAACATAATTACACTGGACAGGAAGAAGAACCAGTAAGAAAGCATATTCATAAAACCGGATGCCATATCACGGGCTCCGATTTGAAGAGGTATTAGGAAGTTACTGAAGGTACCACTGAGGCCGGCTGTAAGTACAAAAAACACCATGATGGTTCCATGCATCGTCACCAGCGCCAGATAAAACTCAGTATCGATTTTTCCTTCCGGCGTTATCCAACCTCCAAGCAATGGCTTTAACCAAGCCAGGTCTGCCTCTGGAAAACCCAGTTGAAGA
>NODE_13772_length_529_cov_2.354430
GCCAGAAAATCATGTAGCTCGAAATAATAGTCACAAAGAAGTGAATATAGCCCAGCGTGTTGTTCATATAGCGGCCATACATTTTAGGAAACCAGTGATAGATGCCCGCAAACATTCCGAAGAAAGCAGCCAGACCCATCACAATATGGAAATGCGCCACCACAAAATACGTGTCGTGCAGTTGAATATCAATGGTAGAGTTTCCTAAGAAAAAACCTGTTGCCGCGCCATATGGTGGCAATCCAGTTGAACACTTTAATAGCCGAAGGAACTGCAATCAACAGCGTAAACAATACGAAAATTGAAGCCACAAACGGGTTCAGCCCGGTAATGAACATGTGGTGTGCCCACACAATAAACGACAGGATAACGATACCAAGGATAGAGAATATCATCGCCTTATAACCAAACACCGGTTTGCGTGCATTTACCGAAAGCACTTCGGACACCAGACCCATTGCAGGAAGAATGATGATGTACACCTCAGGGTGACCTAAGAACCAGAACAGGTGCTGGAACAGAATAGGAC
>NODE_25824_length_412_cov_2.232493
TAAACAAGAAAATAAAACCTAATGACCATAATAACGAAGGGTTAGGATATAGTTGTGCCCCGTACAAAGTCCCTAATCAACTGAAAATTTTGATTCCTGTTGGAACAGCAATAATTATAGTCGCAGCAGTAAAATAGGCTCGGGTATCAACGTCTATCCCTACTGTAAATATATGGTGAGCTCATACAATGAATCCTAAAAGACCGATTGCTAGTATAGCATAAATTATTCCTAAAGCCCCAAATGTCTGTGCTTTACCTCTTTGGTGTGTTGTAATATGAGAGATTATGCCGAATCCAGGTAGGATCAAAATATACACTTCAGGGTGACCAAAGAACCAAAATAGATGTTGGTAAAGAATAGGGTCACCCCCTCCTGCAGGGTCAAAAAATGTAGTATTAAGATTACGATC
>NODE_97631_length_262_cov_0.927536
TTGCCTGGGTTTGGCATTGTTTCGCACATTGTATCTACTTTTAGCCAAAAACAAGTTTTTGGTTATCTAGGCATGGTTTACGCAATGTTGTCGATTGGGATTTTGGGATTTATCGTTTGGGCACATCATATGTATACGGTAGGTCTTGATGTTGATACTCGTGCATACTTTACGGCAGCAACTATAATTATTGCTGTGCCCACTGGTATTAAAGTATTTAGTTGGCTTGCTACAATATGGGGCGGTTCCGTTCATCTAAGAG
>NODE_101116_length_260_cov_0.936585
GGATCCGCCTTCGTCGATGAAGGGGTGGGTACCGGCTGGACGGTGTATCCACCGCTGTCGCAGATCACTTCGCATCCGGGCATGTCGGTGGATATGGGGATTTTCGCCCTGCACCTGGCCGGGATTTCGTCTATTTTGGGCGCCATCAATTTCATCGTTACCGTGTTTAACATGCGGGCGCCGGGCCTGACCTTGTTTAAGATGCCGCTATTCCCCTGGGCGATTCTGATTACCGCGTTCCTGCTGCTGCTTGCGCTTCC
